# Lactic Acid Bacterium Population Dynamics in Artisan Sourdoughs Over One Year of Daily Propagations Is Mainly Driven by Flour Microbiota and Nutrients

**DOI:** 10.3389/fmicb.2018.01984

**Published:** 2018-08-27

**Authors:** Fabio Minervini, Francesca R. Dinardo, Giuseppe Celano, Maria De Angelis, Marco Gobbetti

**Affiliations:** ^1^Department of Soil, Plant and Food Sciences, Università degli Studi di Bari Aldo Moro, Bari, Italy; ^2^Faculty of Science and Technology, Free University of Bozen, Bolzano, Italy

**Keywords:** bakery, traditional, sourdough, flour, carbohydrates, free amino acids, lactic acid bacteria, strains

## Abstract

This study aimed to: (i) assess at what extent traditional, daily propagated, sourdough can be considered a stable microbial ecosystem; (ii) ascertain the drivers of stability/variability. For this purpose, samples of sourdough, flour and environment were collected over 1 year from three different bakeries located in Altamura, Castellana Grotte, and Matera. Culture-dependent and –independent analyses were carried out on all the samples. In addition, sourdough and flour were subjected to biochemical characterization. In all the sourdoughs sampled at the same bakery, cell density of lactic acid bacteria fluctuated of one-two log cycles. However, 16S metagenetic analysis showed that sourdough bacterial microbiota was remarkably stable, in terms of species. Yet, some differences were found during time at intra-specific level. Indeed, bacterial strains succeeded in a 1-year lapse of time or even in 6-months, such as in the case of strains isolated from Altamura sourdough samples. Residual carbohydrates, lactic acid, ethanol and free amino acids varied in the same sourdough collected at different sampling times. These variations could be attributed to combination of various factors, such as fermentation temperature and strain succession. In addition, concentration of flour nutrients varied over 1 year and, in some cases, in a shorter time lapse. This may have favored certain strains over others. For this reason and also because of its inherent contamination by lactic acid bacteria, we found flour as the major driver of strains succession.

## Introduction

Sourdough consists in a cereal based-matrix, containing viable lactic acid bacteria (LAB) and yeasts, used as leavening agent or baking improver (or both). Although at least three different types of sourdough may be recognized, the highest number of studies has been carried out about type I (*alias* traditional) sourdough, usually obtained by spontaneous fermentation of a firm dough, followed by other (5–10) consecutive fermentation (back-slopping) steps in which the fermented dough is used as inoculum (De Vuyst et al., 2009). Traditional sourdough is a relatively complex ecosystem representing a natural source of microbial diversity, that overall varies depending on time and space. One single sourdough may harbor simple (two microbial species) to rather complex (more than six species) microbial consortia at a given time (Minervini et al., 2014). Heterofermentative lactobacilli, such as *Lactobacillus brevis*, *Lactobacillus plantarum*, *Lactobacillus paralimentarius*, *Lactobacillus rossiae*, and especially *Lactobacillus sanfranciscensis*, usually dominate the bacterial community of sourdough (Gobbetti et al., 2016). Other LAB, belonging to the genera *Enterococcus, Lactococcus, Leuconostoc, Pediococcus, Streptococcus*, and *Weissella* are much less frequently encountered in sourdoughs (De Vuyst et al., 2009). Usually the number of yeast species found in a given sourdough is not higher than two (De Vuyst et al., 2016). Yeast cell density is generally lower than LAB, being 1:100 one of the most common ratios between these populations (Gobbetti et al., 2016). *Saccharomyces cerevisiae*, *Kazachstania exigua*, *Kazachstania humilis* (formerly *Candida humilis*), *Torulaspora delbrueckii*, *Wickerhamomyces anomalus*, and *Pichia kudriavzevii* are the most frequent species dominating the fungal community of sourdough (De Vuyst et al., 2016).

Sourdough microorganisms may origin from flour and other ingredients, or may be deliberately added (De Vuyst et al., 2014). Flour, even when irradiated with γ-rays, is a reservoir of bacteria and yeasts that contaminate sourdough (Celano et al., 2016). Such a contamination is mostly direct in traditional sourdough, because during back-slopping flour is mixed with part of previous sourdough (Gobbetti et al., 2016). *Enterococcus* sp., *Lactobacillus graminis*, *L. plantarum*, *Leuconostoc* sp., *Pediococcus pentosaceus*, and *Weissella cibaria* may be regarded as frequent inhabitants of wheat flour (Corsetti et al., 2007; Celano et al., 2016). One *L. plantarum* strain, previously isolated from wheat endophytic component (Minervini et al., 2015a), was retrieved as a member of the sourdough bacterial population (Minervini et al., 2018). Additional ingredients (e.g., vegetables, sugar, salt), if used, carry naturally contaminating microorganisms (Minervini et al., 2016; Ripari et al., 2016). For instance, metagenetic analysis showed that *Lactobacillus* sp. dominated pears and related maceration water, and baker’s yeast. Other Operational Taxonomic Units (OTUs) found in additional ingredients were *Lactococcus* (baker’s yeast), *Leuconostoc* (fruit) and *Streptococcus* (honey) (Minervini et al., 2016). Besides flour and (eventual) additional ingredients, house microbiota is another source of contaminating microorganisms for sourdough. It may be defined as the microbial consortium steadily or transiently contaminating bakery environment (Scheirlinck et al., 2009). Metabolically active *L. sanfranciscensis* was found on surfaces of storage box and dough mixer of four artisan bakeries, demonstrating that sourdough dominant LAB highly contaminate the house microbiota (Minervini et al., 2015b). A complex interplay between bakery house microbiota, flour and process parameters may cause shifts in the sourdough microbial consortia (Van Kerrebroeck et al., 2017).

This study aimed to: (i) assess at what extent traditional, daily propagated, sourdough can be considered a stable microbial ecosystem; (ii) ascertain the drivers of stability/variability. For this purpose, samples of sourdough, flour and bakery environment were collected from three different bakeries over 1 year and analyzed by culture-dependent and –independent approach. In addition, sourdough and flour were subjected to biochemical characterization.

## Materials and Methods

### Bakeries and Workflow of Traditional Sourdoughs

Three artisan bakeries, located in Southern Italy, were monitored in this study: AM (Altamura, Bari), CG (Castellana Grotte, Bari), and MT (Matera). These bakeries were used to propagate their sourdough by one daily back-slopping, with no addition of baker’s yeast or other starters. **Supplementary Table 1** shows the quantity of flour, sourdough and water, as well as the conditions (time and temperature) of incubation for each sourdough. The three bakeries used tap water from the corresponding regional water-works (**Supplementary Table 2**). The daily back-slopping was performed according to the following workflow: collection of one part of previously fermented sourdough from an “ad hoc” storage box and input in the dough mixer exclusively used for sourdough. After fermentation, bakeries AM and CG adopted resting at 4 °C for 10 and 17 h, respectively. Bakers daily cleaned up storage boxes and dough mixers using coarse sponge and drinkable water and finally wiped the surfaces with vinegar.

### Sampling of Wheat Flours, Bakery Environment and Sourdoughs

**FIGURE 1:**
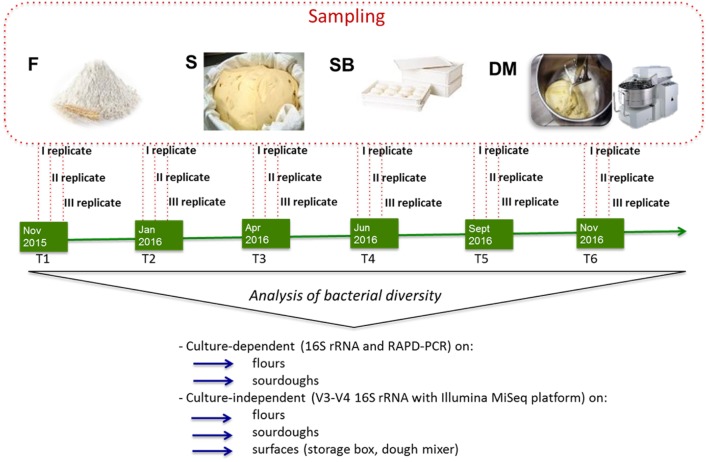
Schematic representation of experimental plan showing the time of sampling (T1, T2, T3, T4, T5, T6), the samples collected (F, flour, S, sourdough, SB, storage box, DM, dough mixer) and the analyses performed on the different samples.

Culture-dependent and –independent microbiological analyses and biochemical analyses were carried out on 20 g of flour. Surfaces of dough mixer and storage box were subjected to swabs for culture-independent analyses and plate counting as previously described (Minervini et al., 2015b). Sourdoughs used by the three bakeries (AM, CG, MT) were collected and transferred to laboratory under refrigerated conditions. Plate count was performed on 10 g of sourdough, within 2 h since sampling. The aliquot (10 g) to be analyzed by culture-independent method was stored at -20°C until analysis. Biochemical analyses were carried out on 10 g of sourdough kept at -20°C. Sampling at bakeries was carried out six times in approximately 1 year (from November 2015 to November 2016). For each bakery and sampling time, flour, surfaces (storage box, dough mixer), and sourdough samples were collected (three replicates) in three different days of the same week (**Figure 1**).

### Enumeration of Bacteria and Yeasts

Presumptive LAB, enterococci, staphylococci, total coliforms, acetic acid bacteria, yeasts and molds were counted using the media (all purchased from Oxoid Ltd., Basingstoke, United Kingdom, except for SDB and Deoxycholate-Mannitol-Sorbitol, which were prepared in laboratory mixing the required ingredients) reported in **Supplementary Table 3**. Prior to plate count, solid samples were mixed with 90 ml of sterile peptone water using a paddle homogenizer (Minervini et al., 2012a; Ercolini et al., 2013). Contaminating microorganisms on the dough mixer and storage box were enumerated after suspending the swabs in saline solution (20 ml). The suspensions were used (directly or after serial dilution) to inoculate plates. Colonies were counted after 48 h of incubation.

### Isolation, Genotypic Characterization and Identification of LAB

Lactic acid bacteria were isolated from sourdoughs and flours sampled at three bakeries at T1 (month 1), T3 (month 6) and T6 (month 12) by randomly selecting at least fifteen colonies (for each replicate) from the SDB plates, as previously described (Minervini et al., 2016). Similarly, LAB were isolated from dough mixer and storage box sampled at three bakeries at T1 and T6. For both these environmental samples, when no presumptive LAB colony was found using SDB, isolation was performed starting from mMRS plates. After extraction of LAB genomic DNA (Ahmed et al., 2009), lactic acid bacterium isolates were biotyped using three primers (P4, P7, and M13) and PCR conditions reported by De Angelis et al. (2001) (P4 and P7) and Siragusa et al. (2009) (M13). The PCR products (3 μl) were separated by electrophoresis at 100 V for 120 min on 1% (w/v) agarose gel containing (0.005%, v/v) GelRed^®^ Nucleic Acid Gel Stain (Biotium, Inc., Fremont, CA, United States), for detecting the DNA by UV transillumination. The molecular sizes of the amplified DNA fragments were estimated by comparison with the HyperLadder^TM^ 1 kb (Bioline Reagents Limited, London, United Kingdom). RAPD-PCR profiles were acquired by Gel Doc EQ System (Bio-Rad, Hercules, CA, United States) and compared using Fingerprinting II InformatixTM Software (Bio-Rad). The reproducibility of RAPD fingerprints was tested as previously described (Siragusa et al., 2009). RAPD fingerprints of the isolates from one biological replicate were the same as the isolates from two other replicates. Therefore, further analysis was carried out just on bacterial strains isolated from one single replicate.

The 16S rDNA gene fragment of LAB was amplified using primers LacbF/LacbR (Corsetti et al., 2004). In addition, three couples of primers were used to amplify the 16S/23S spacer regions which allow discriminating between *Lactobacillus curvatus*, *Lactobacillus fuchuensis*, *Lactobacillus graminis*, and *Lactobacillus sakei* (Berthier and Ehrlich, 1998). Acid production from amygdalin, arbutin, starch, D-tagatose and D-xylose was used as test for excluding the attribution of isolates to *L. fuchuensis* (Sakala et al., 2002). Primers targeting the *recN* gene were used to discriminate species within the *Weissella* genus (Lee et al., 2011). After purification with NucleoSpin Gel and PCR Clean-up (Macherey-Nagel, Germany), partial sequences of 16S rDNA were obtained by Eurofins Genomics (Ebersberg, Germany). The NCBI nucleotide database and BLAST (Altschul et al., 1990) were used to perform pair-wise alignments of sequences.

### Extraction of Total Bacterial DNA

Before extracting total bacterial DNA from flour, the latter (22.7 g) was mixed with filtered demineralized water (27.3 g) to give 50 g of dough. Five grams of dough or sourdough were mixed with 45 ml of saline solution and subjected to 3-min long homogenization in a BagMixer 400P (Interscience, Saint Nom, France). After two centrifugation steps (Cavallo et al., 2017), the microbial pellet was suspended in 2 ml of saline solution and added with 200 mg of glass beads (Sigma–Aldrich, United States) (Lattanzi et al., 2013). The microbial suspension was then moved into ice-bath and sonicated using a Vibra-Cell sonicator (Sonic and Materials Inc., Danbury, CT, United States) equipped with a micro-tip and set at 0.6 Amp for 15 min (3 cycles of 5 min each, with a 5-min-long pause between cycles). After sonication, 500 μl of microbial suspension was treated by the FastPrep Instrument model BIO 101 (Savant Instruments, Hyderabad, India) and DNA was extracted by using the FastDNA Spin Kit for Soil (MP Biomedicals, Illkrich, France), according to the manufacturer’s instructions (Lattanzi et al., 2013).

For extracting total bacterial DNA from surface swabs, 1.25 ml of sample from dough mixer were mixed with 1.25 ml of sample from storage box. The mixture was centrifuged (27670 × *g*, 10 min, 4°C) and the recovered pellet was suspended in 2 ml of sodium phosphate buffer included in the FastDNA Spin Kit for Soil and further treated according to the manufacturer’s instructions.

### Analysis of Bacterial Diversity

16S metagenetics were carried out at BMR Genomics (Padova, Italy), by using the Illumina MiSeq platform. The V3-V4 region of the 16S rRNA gene was amplified using the primers Pro341F and Pro805R (Takahashi et al., 2014) for assessing bacterial diversity. BMR Genomics’ protocols were followed for PCR and sequencing analyses.

The sequenced forward and reverse reads were merged together using the FLASH v1.2.11 software and filtered, with the aim to get sequences with quality higher than 30. OTUs were clustered on the basis of the pick_closed_reference_otus.py method provided by Qiime v 1.9.1 (Caporaso et al., 2010). Taxonomic attribution was carried out searching in the NCBI 16S ribosomal RNA sequences database through BLAST (Altschul et al., 1990).

Relative abundance was calculated for comparing the different samples (Andreotti et al., 2011). Qiime was used to calculate the number of observed species, Chao1 richness index (Chao and Bunge, 2002) and Shannon diversity index (Shannon and Weaver, 1949).

### Determination of Carbohydrates, Organic Acids, Ethanol and Free Amino Acids

Carbohydrates and fermentation metabolites were extracted from a homogeneous suspension of flour or sourdough (5 g) and H_2_SO_4_ 10 mM (45 ml) (Minervini et al., 2012a). Glucose, fructose, maltose, sucrose, lactic acid (only sourdough samples), acetic acid (only sourdough samples) and ethanol (only sourdough samples) were quantified using enzymatic Assay Kits (Megazyme International Ireland Limited, Bray, Co. Wicklow, Ireland), following the manufacturer’s instructions. A second water-soluble extract was prepared for analysis of free amino acids (FAA), using Tris-HCl 50 mM pH 8.8 as homogenizing solution (Minervini et al., 2012a), and analyzing the water-soluble extract through the Biochrom 30 Amino Acid Analyser (Biochrom LTD, Cambridge, United Kingdom) (De Angelis et al., 2007).

### Statistical Analyses

One-way ANOVA was applied to the data from three biological replicates (analyzed twice) and Tukey’s procedure (at *p* < 0.05) was used to perform pair comparison of treatment means. In addition, the data were subjected to permutation analysis using PermutMatrix (Caraux and Pinloche, 2005) and Principal Components Analysis (PCA) using Statistica 7.0 for Windows. Spearman correlations among microbiological (LAB cell density, number of LAB strains per species, relative abundance of LAB OTU), biochemical (pH, concentrations of lactic acid, acetic acid and ethanol) and process (temperature of sourdough fermentation) parameters of sourdoughs sampled at T1, T3, and T6, and concentrations of fermentable carbohydrates and individual FAA in flour sampled at T1, T3 and T6 were computed using Statistica v. 7.0. In addition, for each sampling time, correlations between pH and fermentation temperature against LAB cell density, concentrations of lactic acid, acetic acid, ethanol, total FAA, and residual carbohydrates in sourdoughs were calculated.

### Nucleotide Sequence Accession Number

The 16S rRNA gene sequences are available in the Sequence Read Archive of NCBI (accession number PRJNA478854).

## Results

### Microbial Community of Flour, Bakery Environment and Sourdough Described by Culture-Dependent Method

In the flours analyzed during time (at 1, 3, 6, 8, 10, 12 months), the cell density of presumptive LAB enumerated on mMRS and SDB, ranged from ca. 1.3 to 4.0 and from ca. 2.5 to 4.7 log cfu g^-1^, respectively (**Table 1**). Enterococci varied from ca. 2.1 to 4.1 log cfu g^-1^, but were not detected at T2 (CG), T3 (CG and MT), T4 and T5 (MT). Other common bacterial contaminants found in flour samples were staphylococci and coliforms (**Supplementary Table 4**). Among fungi, yeasts were ever found (1.3 – 4.7 log cfu g^-1^), except for the samples collected in AM and CG at T2 (**Table 1**). Molds represented other occasional fungal contaminants of flour (**Supplementary Table 4**).

**Table 1:** Cell numbers^a^ of presumptive lactic acid bacteria (LAB) and yeasts in the flours (F), sourdoughs (S) (expressed as log cfu/g), and bakery environment (surfaces of dough mixer, DM, and storage box, SB) (expressed as log CFU/cm^2^) sampled in Altamura, Castellana Grotte, and Matera every 2 months.

Sampling time and sample code	LAB on mMRS	LAB on SDB	Enterococci	Yeasts
**Altamura**				
T1-F	3.4 ± 0.0d	4.3 ± 0.3d	3.5 ± 0.9cd	3.0 ± 1.0d
T1-S	6.9 ± 0.7b	7.1 ± 0.5bc	3.5 ± 0.3cd	6.9 ± 0.3a
T1-DM	2.1 ± 1.5e	1.6 ± 0.7f	<0.25	1.7 ± 1.5ef
T1 SB	2.3 ± 0.4e	2.8 ± 0.5e	<0.25	2.0 ± 0.4e
T2-F	2.3 ± 0.6e	3.2 ± 0.9e	2.7 ± 0.0e	< 1
T2-S	7.1 ± 0.1b	6.8 ± 0.2c	4.2 ± 0.7b	6.5 ± 0.3ab
T2-DM	<0.025	<0.025	<0.25	<0.025
T2 SB	1.6 ± 0.4e	1.5 ± 0.3f	<0.25	1.6 ± 0.1f
T3-F	3.5 ± 1.1cd	4.2 ± 1.1de	4.1 ± 1.2bc	3.6 ± 0.0c
T3-S	7.3 ± 0.5ab	7.8 ± 1.1ab	5.3 ± 0.0a	6.3 ± 0.0b
T3-DM	1.7 ± 0.0e	2.5 ± 1.5ef	<0.25	<0.025
T3 SB	2.1 ± 1.3e	3.3 ± 0.3e	<0.25	0.5 ± 0.2g
T4-F	2.3 ± 0.6e	4.5 ± 0.0d	2.5 ± 0.0f	2.2 ± 0.2e
T4-S	7.1 ± 0.1b	7.5 ± 0.5ab	4.5 ± 0.0b	6.9 ± 0.5a
T4-DM	0.3 ± 0.0g	0.7 ± 0.0g	<0.25	<0.025
T4 SB	1.4 ± 0.5ef	1.6 ± 0.9f	<0.25	1.6 ± 0.4f
T5-F	4.0 ± 0.1c	4.7 ± 0.1d	3.2 ± 0.5d	2.5 ± 0.3de
T5-S	7.5 ± 0.5ab	8.1 ± 0.7a	3.4 ± 0.1d	6.5 ± 0.3ab
T5-DM	1.0 ± 0.1f	1.0 ± 0.4fg	<0.25	2.0 ± 0.5e
T5 SB	1.7 ± 0.7e	2.7 ± 0.7e	<0.25	1.9 ± 0.6e
T6-F	2.0 ± 0.3e	4.4 ± 0.0d	3.2 ± 0.4d	1.7 ± 0.3f
T6-S	7.7 ± 0.1a	8.0 ± 0.2a	2.0 ± 0.0g	6.5 ± 0.0a
T6-DM	0.5 ± 0.1g	<0.025	<0.25	< 0.025
T6 SB	0.3 ± 0.0g	1.1 ± 0.0f	< 0.25	<0.025
**Castellana Grotte**				
T1-F	2.9 ± 0.4ef	4.0 ± 0.6d	2.7 ± 0.2e	3.3 ± 1.5def
T1-S	7.1 ± 0.3a	8.2 ± 0.5a	4.7 ± 0.5b	6.8 ± 0.5a
T1-DM	0.5 ± 0.3j	0.9 ± 0.1i	1.4 ± 0.0f	1.6 ± 0.4gh
T1 SB	1.0 ± 0.4hi	1.5 ± 0.0h	2.0 ± 0.5e	2.0 ± 0.3g
T2-F	1.7 ± 0.0g	3.0 ± 0.4e	<2	<1
T2-S	4.9 ± 0.0d	7.9 ± 0.1a	3.5 ± 0.5d	6.7 ± 0.5ab
T2-DM	<0.025	<0.025	<0.25	0.8 ± 0.6hij
T2 SB	0.7 ± 0.6ij	0.7 ± 0.6hi	<0.25	1.1 ± 0.8ghi
T3-F	1.3 ± 0.0h	3.2 ± 0.4e	<2	3.3 ± 0.0e
T3-S	7.2 ± 0.5a	7.5 ± 1.0ab	5.6 ± 0.7a	6.9 ± 0.5a
T3-DM	<0.025	3.0 ± 1.0def	<0.25	<0.025
T3 SB	3.2 ± 0.6e	3.3 ± 0.5de	2.1 ± 1.8def	0.6 ± 0.2j
T4-F	2.0 ± 0.4f	3.8 ± 0.2d	2.6 ± 0.0e	3.0 ± 0.7ef
T4-S	4.8 ± 0.0d	7.5 ± 0.1b	4.0 ± 0.3cd	6.3 ± 0.1b
T4-DM	<0.025	<0.025	<0.25	<0.025
T4 SB	3.5 ± 0.5e	3.8 ± 0.2d	1.1 ± 0.0gh	1.9 ± 0.6g
T5-F	1.7 ± 0.0g	2.5 ± 0.0f	2.3 ± 0.0e	1.3 ± 0.0h
T5-S	5.4 ± 0.1c	7.0 ± 0.0c	4.3 ± 0.1c	5.4 ± 0.8c
T5-DM	<0.025	<0.025	<0.25	<0.025
T5 SB	1.5 ± 1.3gh	0.8 ± 0.5hi	1.1 ± 0.0gh	0.9 ± 0.3ij
T6-F	2.5 ± 0.4f	3.1 ± 0.2e	2.1 ± 0.2e	2.7 ± 0.0f
T6-S	6.5 ± 0.1b	7.0 ± 0.9bc	4.2 ± 0.7bc	4.3 ± 0.5d
T6-DM	<0.025	<0.025	0.8 ± 0.0hi	1.1 ± 0.0h
T6 SB	1.9 ± 0.5fg	1.8 ± 0.7g	0.6 ± 0.0i	1.4 ± 0.1h
**Matera**				
T1-F	3.2 ± 0.3d	4.0 ± 0.2c	2.9 ± 0.8cde	4.7 ± 0.2d
T1-S	6.9 ± 1.8abc	7.5 ± 1.2a	4.9 ± 0.2a	6.8 ± 0.4c
T1-DM	0.3 ± 0.0j	2.2 ± 0.0e	<0.25	1.9 ± 1.8efg
T1 SB	1.2 ± 0.2h	1.8 ± 1.4de	<0.25	2.5 ± 1.2e
T2-F	2.7 ± 0.2e	3.6 ± 0.6cd	3.2 ± 0.0d	2.7 ± 0.5e
T2-S	7.5 ± 0.0a	7.1 ± 0.5b	3.9 ± 1.0bc	7.8 ± 0.1b
T2-DM	<0.025	1.8 ± 0.0e	1.7 ± 0.0f	1.2 ± 0.0gh
T2 SB	1.3 ± 0.0h	1.7 ± 1.0de	<0.25	1.2 ± 1.0fghi
T3-F	2.4 ± 0.3f	3.8 ± 0.5cd	<2	3.3 ± 1.7de
T3-S	7.3 ± 0.4a	6.7 ± 1.4ab	4.9 ± 0.0a	8.2 ± 0.4a
T3-DM	<0.025	3.6 ± 0.0c	<0.25	<0.025
T3 SB	1.1 ± 0.4h	2.6 ± 1.1cde	<0.25	2.5 ± 0.6e
T4-F	3.1 ± 0.4de	3.1 ± 0.7d	<2	1.9 ± 0.0f
T4-S	6.6 ± 0.2b	7.3 ± 0.5ab	4.6 ± 0.9ab	7.8 ± 0.1b
T4-DM	1.6 ± 0.0g	1.7 ± 0.0e	<0.25	1.2 ± 0.7fg
T4 SB	0.4 ± 0.0ij	2.4 ± 1.8cde	<0.25	0.4 ± 0.1i
T5-F	2.7 ± 0.4ef	4.3 ± 0.2c	<2	2.1 ± 0.0e
T5-S	6.2 ± 0.8bc	7.3 ± 0.5ab	4.1 ± 0.2b	7.9 ± 0.1b
T5-DM	<0.025	1.2 ± 0.0f	<0.25	<0.025
T5 SB	0.6 ± 0.0i	1.2 ± 0.0f	<0.25	1.5 ± 0.0gh
T6-F	2.6 ± 0.6def	3.9 ± 0.1c	2.1 ± 0.7e	2.2 ± 1.2ef
T6-S	5.5 ± 0.0c	7.9 ± 1.2a	3.4 ± 0.3cd	8.0 ± 0.0ab
T6-DM	1.8 ± 0.0g	1.8 ± 0.0e	<0.25	1.8 ± 0.0fg
T6 SB	<1	0.6 ± 0.0g	<0.25	1.3 ± 0.0h

*^a^Values (mean of three replicates ± standard deviation) in the same column, within the same bakery, followed by different letters are significantly different (*p* < 0.05)*.

Presumptive LAB were found in AM sourdough at cell density ranging from ca. 6.9 to 7.7 log cfu g^-1^ (count on mMRS) and from ca. 6.8 to 8.1 log cfu g^-1^ (SDB) (**Table 1**). Microbiota of CG sourdough was dominated by LAB, estimated in the ranges 4.8 – 7.1 (mMRS) and 7.0 – 8.2 (SDB) log cfu g^-1^. MT sourdough was characterized by LAB cell densities in the ranges 5.5 – 7.5 (estimated on mMRS) and 6.7 – 7.9 (SDB) log cfu g^-1^. In all the sourdough samples, enterococci and staphylococci ever represented the two main subdominant bacterial groups (**Table 1** and **Supplementary Table 4**). Coliforms and acetic acid bacteria were variously found as occasional contaminants (**Supplementary Table 4**). Yeasts were found in the sourdough samples at steady cell density, except for CG, wherein they decreased starting from T3, down to ca. 4.3 log cfu g^-1^ at T6 (**Table 1**).

Lactic acid bacteria ever contaminated, although at low levels, dough mixer (AM and MT) and storage box (all three bakeries) (**Table 1**). Enterococci represented occasional contaminants (**Table 1**), as well as staphylococci, coliforms and acetic acid bacteria (**Supplementary Table 4**). Yeasts were the most common contaminants in the bakery environment of CG and MT (**Table 1**).

### Lactic Acid Bacterium Biota in Sourdoughs, Flour and Bakery Environment

Gram-positive, catalase-negative, non-motile, cocci and rods able to acidify broth (390, 301, 75, and 121 isolates from sourdough, flour, dough mixer and storage box, respectively) were subjected to RAPD-PCR analysis. For each sourdough and sampling time, all the detected strains were identified. AM sourdough was dominated by *L. sanfranciscensis* (**Table 2**). In this sourdough *Leuconostoc citreum* was found as sub-dominant. Lactic acid bacterium biota of sourdough samples collected in CG was composed of strains belonging to *L. sanfranciscensis* and *Lactobacillus sakei* (detected at all the sampling times), *Ln. citreum* and *Leuconostoc mesenteroides*. *Weissella cibaria* dominated the MT sourdough, whereas *L. sanfranciscensis* was sub-dominant and *P. pentosaceus* was found only at T1. Within the bacterial species isolated from AM sourdough, no strain was found as stable component of the bacterial biota (**Table 2**). CG sourdough sampled at T1 shared only one strain of *L. sanfranciscensis* (CG3) with CG sourdough at T3; however, this strain was not retrieved in the CG sourdough at T6. No strain was shared by the CG sourdough sampled at T3 and T6. Similarly, lactic acid bacterium strains isolated from MT sourdough at T1 were different from those isolated from the same sourdough sampled (after 5 months) at T3. In the latter sample one strain of *W. cibaria* (MT6) was retrieved also 6 months later in the MT sourdough sampled at T6 (**Table 2**).

**Table 2:** Number of LAB isolates for each strain (based on RAPD-PCR genotyping) from flour (F) and sourdough (S) collected at the beginning (T1), in the middle (T3) and at the end (T6) of the monitoring campaign and from dough mixer (DM) and storage box (SB) sampled at the beginning (T1) and end (T6) of the monitoring campaign performed on the bakeries located in Altamura (AM), Castellana Grotte (CG) and Matera (MT).

	T1				T3		T6			
	F	S	DM	SB	F	S	F	S	DM	SB
**AM**					
*Lactobacillus sanfranciscensis* AM1	4	4								
*L. sanfranciscensis* AM2		6								
*L. sanfranciscensis* AM3		5								
*L. sanfranciscensis* AM4		4								
*L. sanfranciscensis* AM5		7								
*L. sanfranciscensis* AM6			3	7						
*Leuconostoc citreum* AM1	27	8	10	15						
*L. sanfranciscensis* AM7					3	8				
*L. sanfranciscensis* AM8					5	24				
*L. sanfranciscensis* AM9						4				
*Ln. citreum* AM2					19	5				
*L. sanfranciscensis* AM10								8		
*L. sanfranciscensis* AM11							5	6		
*L. sanfranciscensis* AM12								2		
*L. sanfranciscensis* AM13								11		
*L. sanfranciscensis* AM14								9		
*L. sanfranciscensis* AM15										3
*Ln. citreum* AM3							24	5		16
**CG**					
*L. sanfranciscensis* CG1		7								
*L. sanfranciscensis* CG2		4								
*L. sanfranciscensis* CG3	4	19				3				
*L. sanfranciscensis* CG4		5								
*L. sanfranciscensis* CG5			4	10						
*Lactobacillus sakei* CG1		2								
*L. sakei* CG2			4	12						
*Ln. citreum* CG1	20	4								
*Leuconostoc mesenteroides* CG1		3								
*L. sanfranciscensis* CG6					6	16				
*L. sanfranciscensis* CG7					4	13				
*L. sakei* CG3						7				
*Ln. citreum* CG2					27					
*L. sanfranciscensis* CG8								8		
*L. sanfranciscensis* CG9								4		
*L. sanfranciscensis* CG10							7	15		
**MT**					
*L. sanfranciscensis* MT1	5	3								
*L. sanfranciscensis* MT2			3	3						
*Weissella cibaria* MT1	23	13	15	10						
*W. cibaria* MT2		4		11						
*W. cibaria* MT3		2	4							
*W. cibaria* MT4		5								
*Pediococcus pentosaceus* MT1	2	11								
*L. sanfranciscensis* MT3					5	3				
*W. cibaria* MT5					7	8				
*W. cibaria* MT6					11	24	9	15	8	3
*W. cibaria* MT7					9	5				
*L. sanfranciscensis* MT4							7	3		
*L. sanfranciscensis* MT5									4	
*W. cibaria* MT8								14		
*W. cibaria* MT9							9	11	5	
*W. cibaria* MT10							9		8	
*W. cibaria* MT6					13	19	12	13	7	3

*Lactobacillus sanfranciscensis* was isolated from all the flours and environmental (dough mixer and storage box) samples. At all the sampling times at least one strain of *L. sanfranciscensis* isolated from the flour was also retrieved in the sourdough. In addition, *Ln. citreum* was isolated from flours sampled in AM and CG. Almost all the strains of *Ln. citreum* isolated from flour were also found in the sourdough of the bakeries using that flour. In addition, *Ln. citreum* was also isolated from AM bakery environment. *L. sakei* was found in the environmental samples collected in CG. Sourdough and environmental samples collected at CG bakery shared some strains of *L. sakei*. Regarding the MT bakery, *W. cibaria* was retrieved both in flour and environmental samples and in some cases strains inhabiting these ecosystems were also found in the MT sourdough (**Table 2**).

### Microbial Community of Flour, Bakery Environment and Sourdough Described by 16S Metagenetic Analysis

The bacterial diversity of flour, bakery environment (dough mixer, storage box) and sourdough was described by 16S metagenetic analysis. Environmental samples from AM and MT bakeries gave no PCR product and therefore were not analyzed any further. A total of 3,452,547 high quality sequences of 16S rRNA gene amplicons were obtained, with an average of about 67,700 sequences per sample and an average sequence length of 476 bp. Good’s estimated sample coverage was ever higher than 99.9% (data not shown). The environmental samples from CG bakery showed the highest alpha diversity values (**Table 3**). As expected, higher (*p* < 0.05) alpha diversity values were found in flour than in sourdough, the only exception being the sourdough collected at month 6 (T3) in MT bakery. All the flours showed no significant (*p* > 0.05) differences during time in terms of number of OTUs and Chao1 index. Shannon index varied (*p* < 0.05) during time for CG and MT flours. During time AM flours showed not significantly (*p* > 0.05) different values of Shannon index. The AM sourdough showed an increasing (*p* < 0.05) number of observed OTUs and Shannon index passing from T1 to T6 (**Table 3**). On the opposite, the CG sourdough sampled at month 6 (T3) was characterized by the highest (*p* < 0.05) number of OTUs and Shannon index, followed by the one sampled at T1. MT sourdough was characterized by increased (*p* < 0.05) alpha diversity indices when comparing T3 with T1; then, Chao1 and Shannon indices at T6 did not significantly (*p* > 0.05) differ from those found at T1.

**Table 3:** Alpha diversity indexes^a^ of *Bacteria* (16S rRNA) found in the flours (F) collected in Altamura (AM), Castellana Grotte (CG) and Matera (MT), found in the swab samples of bakery equipment (E) collected in CG at the beginning (T1) and end (T6) of the monitoring campaign and found in the sourdoughs (S) collected in AM, CG and MT at the beginning (T1), in the middle (T3) and at the end (T6) of the monitoring campaign.

Sample	Observed species	Chao1	Shannon
AM_F_T1	89.00 2.65c	103.65 10.92b	3.19 0.01cd
AM_S_T1	18.00 ± 3.61h	29.28 ± 17.53cd	0.16 ± 0.01k
AM_S_T3	23.00 ± 2.64g	27.45 ± 5.80cd	0.42 ± 0.07i
AM_F_T6	87.67 ± 2.52c	97.29 ± 7.05b	3.18 ± 0.02cd
AM_S_T6	44.67 ± 4.51d	56.42 ± 7.78c	0.62 ± 0.08h
CG_F_T1	79.33 ± 4.51c	89.35 ± 10.89b	2.75 ± 0.03f
CG_S_T1	34.67 ± 0.58e	45.64 ± 7.33cd	0.44 ± 0.01i
CG_E_T1	216.00 ± 1.73b	240.57 ± 11.96a	6.37 ± 0.01a
CG_S_T3	43.33 ± 3.05d	48.12 ± 6.40cd	0.78 ± 0.08h
CG_F_T6	86.67 ± 3.06c	96.13 ± 10.62b	2.93 ± 0.04e
CG_S_T6	18.67 ± 1.15h	23.56 ± 5.05d	0.20 ± 0.01k
CG_E_T6	230.33 ± 4.16a	233.47 ± 2.99a	4.71 ± 0.00b
MT_F_T1	88.00 ± 1.00c	93.65 ± 4.95b	3.31 ± 0.01c
MT_S_T1	24.67 ± 1.53g	34.00 ± 4.00cd	0.39 ± 0.03ij
MT_S_T3	80.33 ± 0.58c	80.58 ± 1.01b	1.68 ± 0.03g
MT_F_T6	84.67 ± 3.21c	90.11 ± 5.55b	3.02 ± 0.02de
MT_S_T6	30.00 ± 1.00f	59.50 ± 27.88c	0.38 ± 0.02j

^a^*Values (mean of three replicates ± standard deviation) in the same column followed by different letters are significantly different (*p* < 0.05)*.

**FIGURE 2:**
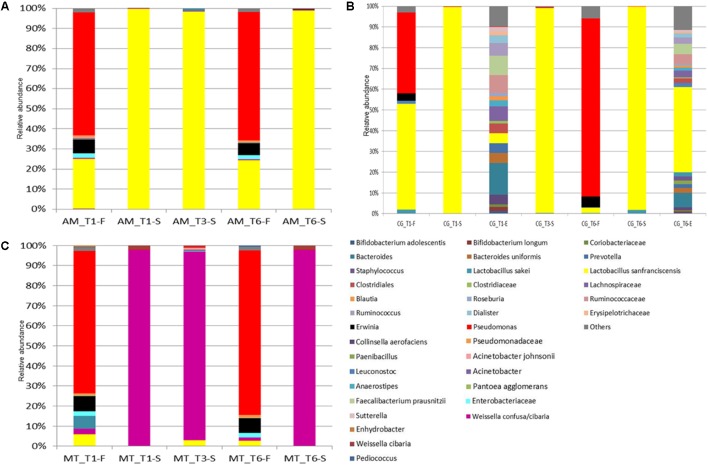
Relative abundance (%) of bacterial OTUs classified at the highest possible taxonomic level (species/genus/family) found in the flour (F), sourdough (S), environment (E) sampled at Altamura **(A)**, Castellana Grotte **(B)**, and Matera **(C)** bakeries at month 1 (T1), 6 (T3), and 12 (T6). Only OTUs with a relative abundance ≥ 0.1% in at least one sample for each bakery are shown.

A core bacterial microbiota was shared by all the flours. Overall, *Pseudomonas* sp. was the most abundant bacterial OTU (minimum relative abundance of ca. 35%) (**Figure 2** and **Supplementary Tables 5**–**7**). The only exception was found in the flour sampled in CG bakery at T1, wherein the most abundant OTU was *L. sanfranciscensis*. The latter was found at relative abundances of ca. 24% in the AM flour, and at even lower abundance in the flours sampled in MT (T1 and T6) and CG (T6). *Erwinia* sp. was also detected (3.7 – 7.6%) in all the flour samples.

Castellana Grotte bakery environment showed a qualitatively steady bacterial contamination during time (**Figure 2** and **Supplementary Table 6**). *Bacteroides* sp., *L. sanfranciscensis*, *Clostridiales*, *Lachnospiraceae*, *Ruminococcaceae*, *Faecalibacterium prausnitzii* and *Ruminococcus* sp. were among the most abundant bacterial OTUs detected. In detail, *L. sanfranciscensis* was found in the environmental sample collected at T6 at a relative abundance of ca. 40%, much higher (*p* < 0.05) than in the sample collected at T1.

As expected, *L. sanfranciscensis* dominated (≥98% of relative abundance) the bacterial microbiota of the sourdoughs sampled in AM and CG (**Figure 2** and **Supplementary Tables 5**, **6**). This species was also detected (0.01 – 2.83%) in the MT sourdoughs, which were dominated by *Weissella confusa*/*cibaria* (**Figure 2** and **Supplementary Table 7**). Other OTUs variously found in the sourdough samples, at relative abundances lower than 2% were: *Leuconostoc* sp. (AM and CG), *W. confusa*/*cibaria* (AM), and *L. sakei* (CG).

### Biochemical Features of Flours and Sourdoughs

AM and MT flours (durum wheat) contained higher (*p* < 0.05) concentration of sucrose, but lower (*p* < 0.05) glucose than CG flour (soft wheat) (**Table 4**). In addition, maltose was higher (*p* < 0.05) in MT than in CG flour. No significant (*p* > 0.05) difference was found for any fermentable carbohydrate between AM and MT flours. Glucose concentration in flours sampled at MT and, especially, CG was extremely variable during time (**Supplementary Figure 1A** and **Supplementary Table 8**). Fructose concentration was extremely variable in CG flour (**Supplementary Figure 1B** and **Supplementary Table 8**). Overall sucrose concentration was variable during time, especially for MT flours (**Supplementary Figure 1C** and **Supplementary Table 8**). Concentration of maltose extremely varied during time, especially for AM flour (**Supplementary Figure 1D** and **Supplementary Table 8**). Concentration of individual and total FAA varied in the flours depending on the bakery and time of sampling (**Supplementary Table 8**).

**Table 4:** Concentration (mM) of fermentable carbohydrates in flours.

	Glucose			Fructose			Sucrose			Maltose		
	AM	CG	MT	AM	CG	MT	AM	GC	MT	AM	CG	MT
	7.17^a^	11.83	7.17	7.17	12.17	7.83	7.83	2.83	8.83	28.83	19.33	34.67
**Group Student’s *t*-test *P*-values**					
AM vs. CG	0.03			0.06			0.00			0.13		
AM vs. MT	1.00			0.16			0.51			0.36		
CG vs. MT	0.03			0.09			0.00			0.01		

*^a^Mean values of three replicates*.

**FIGURE 3:**
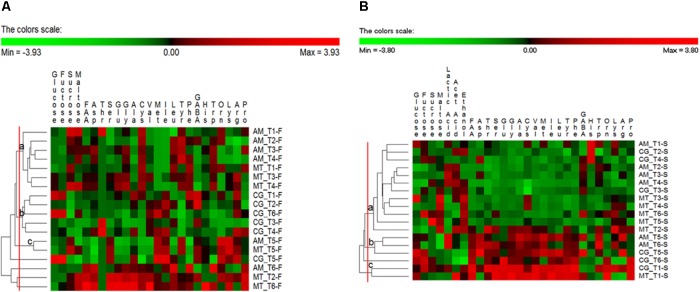
Permutation analysis based on concentrations of glucose, fructose, sucrose, maltose, lactic acid, acetic acid, ethanol, total (FAA) and individual free amino acids in the flours **(A)** and sourdoughs **(B)** collected at Altamura (AM), Castellana Grotte (CG), and Matera (MT) during time. Euclidean distance and McQuitty’s criterion were used for clustering. Colors correspond to normalized mean data levels from low (green) to high (red). The color scale, in terms of units of standard deviation, is also shown.

Permutation analysis was used to assess changes in flour substrates (fermentable carbohydrates and FAA) between bakeries and sampling times (**Figure 3A**). Flour samples collected at AM from T1 (month 1) to T4 (month 8) were grouped in the cluster a, together with three flour samples collected in MT (at T1, T3, and T4). These samples were characterized by low concentration of glucose, fructose, ser, val, ile and met. At month 10 (T5), the flours collected at these two bakeries were clustered again (cluster c), because of relatively high concentrations of sucrose, ser, met, ile, tyr and lys. All the flours collected in CG were grouped in the cluster b, except for the one collected at T5 (unclustered). These samples were characterized by low concentrations of sucrose and FAA, especially ser, gly, leu, trp, and arg (**Figure 3A**).

As concerns the sourdough samples, maltose was the residual fermentable carbohydrate found at highest concentration, followed by glucose and fructose. Sourdough samples collected at AM showed highly variable concentrations of all carbohydrates, especially glucose, during time (**Supplementary Figure 1** and **Supplementary Table 9**). Glucose was the highest variable carbohydrate found in CG sourdough samples, followed by fructose. MT sourdough was characterized by high variation of fructose, maltose and, especially, glucose during time (**Supplementary Figure 1** and **Supplementary Table 9**). Concentration of individual and total FAA varied depending on the bakery and sampling time (**Supplementary Table 9**). Sourdough samples collected in AM showed quite variable concentrations of lactic acid during time, whereas lower variations were found for ethanol and, especially, acetic acid (**Supplementary Figure 2** and **Supplementary Table 9**). The pH of this sourdough varied from ca. 3.75 to 4.13 (3.98 on average) (**Supplementary Table 9**). CG sourdough (having pH varying from ca. 3.90 to 4.57; 4.36 on average) was characterized by high variation in ethanol, whereas organic acids varied at much lesser extent (**Supplementary Figure 2** and **Supplementary Table 9**). The only extreme value for this sourdough was found at T1, characterized by high concentration of lactic acid and in agreement with the lowest pH found. Overall, samples of sourdough collected in MT showed little variations of fermentation metabolites during time, despite the changing concentrations of maltose and sucrose. The pH ranged from ca. 4.47 to 4.80, being on average 4.65 (**Supplementary Table 9**).

Permutation analysis was used also for substrates and fermentation metabolites found in the sourdough samples (**Figure 3B**). A large cluster (a) grouped together sourdough samples generally sharing relatively low concentrations of FAA. All the samples collected in MT fell in this cluster, except for the one sampled at T1, which was grouped (cluster c) with the sourdough collected at CG at the same sampling time. These two samples were characterized by high concentrations of ethanol and most of the individual FAA. Another small cluster (b) included the sourdoughs sampled at T5 (AM and CG) and T6 (AM), which shared low concentrations of sucrose, maltose, ethanol, his, orn and pro and high concentrations of many FAA, especially asp, val, met and trp (**Figure 3B**).

**FIGURE 4:**
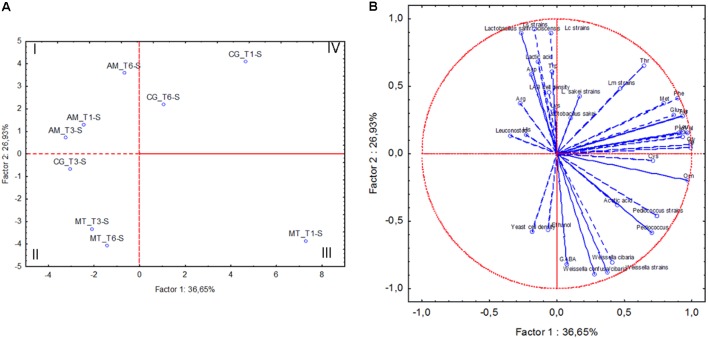
Score **(A)** and loading **(B)** plots of first and second principal components after Principal Component Analysis based on cell density of lactic acid bacteria (LAB) and yeasts, concentrations of lactic acid, acetic acid, ethanol, individual free amino acids, percentage of relative abundance of lactic acid bacterium OTUs, number of strains allotted to *Lactobacillus sanfranciscensis* (Ls strains), *Leuconostoc citreum* (Lc strains), *Weissella* sp. (Weissella strains), *Leuconostoc mesenteroides* (Lm strains), *Pediococcus pentosaceus* (Pediococcus strains) and *Lactobacillus sakei* (L. sakei strains) in the sourdoughs collected at Altamura (AM), Castellana Grotte (CG), and Matera (MT) at month 1 (T1), 6 (T3) and 12 (T6).

The following data related to sourdough samples collected at the three bakeries at month 1 (T1), 6 (T3) and 12 (T6) were subjected to Principal Component Analysis (PCA): cell densities of yeasts and LAB, relative abundance of OTU attributed to LAB, number of strains for each isolated lactic acid bacterium species, concentrations of lactic and acetic acid, ethanol and individual FAA. The sourdough samples collected in AM grouped separately from the others, mainly because of high concentration of lactic acid (**Figure 4**). MT sourdough samples collected at T3 and T6 fell in the second quadrant of the plain, sharing higher yeast cell density than the sample collected at T1. The latter (MT_T1-S) was separated from all the others (third quadrant), being characterized by high concentrations of acetic acid and ornithine. High concentrations of phe and pro were shared by the sourdoughs sampled in CG at T1 and T6 (fourth quadrant). The sourdough collected in the same bakery at T3 differed from the two previous samples, being more similar to MT sourdoughs collected at T3 and T6.

### Correlations Between Flours and Sourdough and Between Microbiological, Biochemical and Process Parameters of Sourdoughs

Concentration of lactic acid was positively (*p* < 0.05) correlated with cell density of LAB (enumerated on SDB, *r* = 0.63) and number of strains of *L. sanfranciscensis* (*r* = 0.66) and *Ln. citreum* (*r* = 0.69). The latter species was negatively (*p* < 0.05, *r* = -0.65) correlated with ethanol concentration. The number of *W. cibaria* strains was correlated (*p* < 0.05, *r* = 0.62) with lys in flour. Higher positive correlation (*r* = 0.81) was found between *Ln. mesenteroides* and concentration of GABA in flour. Positive correlations (*p* < 0.05, *r* > 0.77) were also found between the number of *L. sakei* strains and met, fructose and glucose in flour. However this bacterial species was negatively (*p* < 0.05, *r* < -0.70) correlated with concentrations of gly, cys, tyr and trp in flour. In addition, *L. sanfranciscensis* was positively (*p* < 0.05) correlated with temperature of sourdough fermentation (*r* = 0.98 for relative abundance of the OTU and *r* = 0.88 for number of strains). On the contrary, the relative abundance of *Weissella* sp. and the number of strains of *W. cibaria* were negatively (*p* < 0.05) correlated with temperature (*r* = -0.98 and *r* = -0.96, respectively). Weaker negative correlations were found between sourdough pH and relative abundance of *L. sanfranciscensis* (*r* = -0.73) and number of *L. sanfranciscensis* strains (*r* = -0.76). pH and *Ln. citreum* were also negatively (*p* < 0.05) correlated (*r* = -0.75 for relative abundance of the OTU and *r* = -0.76 for number of strains). pH was positively (*p* < 0.05) correlated with relative abundance of *Weissella* (*r* = 0.72) and residual maltose (*r* = 0.74) and negatively (*p* < 0.05) correlated with lactic acid (*r* = -0.92).

In order to elucidate any further influence exerted by fermentation temperature and sourdough pH, correlations were calculated at each sampling time (**Supplementary Table 10**). Both parameters were negatively (*p* < 0.05) correlated; temperature was negatively (*p* < 0.05) correlated with maltose (except for T1) but positively with lactic acid. Since sourdough fermentation temperature, during approximately 1 year, varied by 6.5 and 10°C (for AM and CG, respectively), correlations were calculated within each sourdough monitored during time (**Supplementary Table 11**). For AM sourdough, temperature was positively (*p* < 0.05) correlated with ethanol and residual sucrose; pH was positively (*p* < 0.05) correlated with residual maltose, but negatively with total FAA. For both sourdoughs, negative correlations were found between pH and LAB cell density.

## Discussion

Traditional sourdough is prepared by spontaneous fermentation of cereal flour-based firm dough, followed by daily back-slopping steps wherein the previously fermented dough serves as inoculum. While at the beginning of the first fermentation, Proteobacteria and Bacteroidetes are the main dominant bacterial phyla, subsequently Firmicutes (almost exclusively represented by LAB) become dominant (Ercolini et al., 2013). Within LAB, different genera/species may succeed during preparation. During the first phase (1–2 days of back-slopping) *Enterococcus*, *Lactococcus* and *Leuconostoc* dominate. Then (3–5 days of back-slopping), *Lactobacillus*, *Pediococcus* and *Weissella* outgrow the previous genera. Finally (6–10 days of back-slopping) *L. sanfranciscensis*, *Lactobacillus fermentum* and *L. plantarum* are selected (Van der Meulen et al., 2007; Weckx et al., 2010). The sources of sourdough LAB have been well established, being represented by flour and house microbiota (Gobbetti et al., 2016).

Opinions about stability of sourdough microbiota are divergent. Once sourdough reaches maturity, its typical microbiota could be stable or not (Gobbetti et al., 2016). Overall, several studies highlighted that lactic acid bacterium species inhabiting a given sourdough can be retrieved at different sampling times. For instance, 39 traditional sourdoughs sampled twice a year in 11 Belgian bakeries showed a stable lactic acid bacterium biota, dominated by *L. sanfranciscensis*, *L. paralimentarius*, *L. plantarum* and *Lactobacillus pontis* (Scheirlinck et al., 2008). *Lactobacillus panis*, *Lactobacillus frumenti*, *Lactobacillus amylolyticus* and *Lactobacillus acetotolerans* steadily dominated industrial French sourdoughs during 10 days of propagation (Vera et al., 2012). *Lactobacillus helveticus*, *L. panis* and *L. pontis* were stable during long-time sourdough propagation (Viiard et al., 2012). Even in this frame, it was found that rye sourdoughs propagated at room temperature harbored different ratios of bacterial species, depending on the season of sampling (Viiard et al., 2016).

Much more controversy exists when sourdough stability is considered in terms of bacterial strains. One reutericyclin-producing strain of *Lactobacillus reuteri* persisted for years in German rye sourdough (Gänzle and Vogel, 2003). On the other hand, some strains of *L. sanfranciscensis* and *L. plantarum* were outgrown by flour autochthonous LAB in just 10 days of propagation (Siragusa et al., 2009; Minervini et al., 2010). Although there is uncertainty about the stability of microbial strains in sourdough, it would seem that this feature is affected by flour and house microbiota. If these two drivers were stable, sourdough microbiota would probably be stable too (Gobbetti et al., 2016).

In the current study, three bakeries applying sourdough biotechnology for long time were monitored over 1 year, in order to assess the weight of potential drivers on bacterial stability. The bakeries were chosen because they propagated sourdough using different flour (species and cultivars of wheat) and technology parameters (e.g., inoculation ratio, time of fermentation). Over 1 year, each baker used the same type (but different batches) of flour and the same conditions for propagating the sourdough. However, fermentation temperature was fairly different among the three bakeries and also within the AM and CG bakeries. As expected, temperature seemed to affect pH and lactic acid, because relatively high temperatures favor fermentation by LAB. Likewise, when relatively low temperatures were applied, high concentration of maltose remained in sourdough. In addition, within the AM sourdoughs, pH seemed to affect concentration of FAA. Low pH favors activity of flour endogenous proteases and therefore FAA are released at high extent. The negative correlation between pH and FAA could also be explained taking into account that low pH is caused by metabolically active sourdough LAB, which are well-known for their peptidase activities (Gänzle et al., 2008). In all the sourdoughs sampled at the same bakery, cell density of LAB fluctuated of one-two log cycles. However, 16S metagenetic analysis showed that sourdough bacterial microbiota was remarkably stable, in terms of species. *L. sanfranciscensis* was isolated as the dominant species from the sourdough samples collected in CG and AM. The sourdough of the latter bakery had been characterized ca. 5 years ago and harbored the same species at sub-dominant level, being dominated by *Leuconostoc citreum* (Lattanzi et al., 2013). The sourdough samples collected in MT harbored *L. sanfranciscensis* and, especially, *W. cibaria*. The same species, along with other species, although in different proportions, had been previously isolated from the traditional sourdough used at the same bakery (Minervini et al., 2012b). In the current study, culture-dependent and –independent analyses detected *L. sanfranciscensis* and *W. cibaria* also in bakery environmental samples and, especially, in flour. Compared to MT (fermentation temperature: 10°C), AM and CG bakeries applied relatively higher temperatures during back-slopping and this could have favored *L. sanfranciscensis* over *Weissella* sp. and other LAB genera/species, in agreement with Lhomme et al. (2015). Although *L. sanfranciscensis* and *Weissella* sp. are both able to grow at 15°C, the genus *Weissella* has been frequently encountered among the dominant bacteria of food items (e.g., kimchi) fermented at 10 – 15°C (Cho et al., 2006). Thus, prevalence of *Weissella* sp. in MT sourdoughs would result from different factors: (i) it normally contaminated the flour used for back-slopping and MT bakery environment (especially the surface of dough mixer); (ii) its growth was favored by relatively high pH values (4.5 – 4.8) (Van Kerrebroeck et al., 2017); (iii) it adapted at low temperature better than *L. sanfranciscensis*.

Notwithstanding the sourdoughs considered in this study proved to be stable in terms of bacterial species, some differences were found during time at intra-specific level. Indeed, bacterial strains succeeded in a 1-year lapse of time or even in 6-months, such as in the case of strains isolated from AM sourdough samples. Succession of strains in traditional sourdough may occur even during 2–3 months of daily back-slopping (Minervini et al., 2012b). In this study concentrations of residual carbohydrates, lactic acid, ethanol or FAA were subjected to variations depending on the bakery considered, as shown by PCA. In some cases, variations of these biochemical features were found also within the same sourdough collected at different sampling points. Strains succession could have influenced sourdough biochemical features. However, fermentation temperature, by influencing metabolic activity (acid production and release of FAA) and growth of LAB and, through sourdough pH, activity of flour endogenous enzymes, affected some biochemical features. This result is in agreement with a previous study that showed that high concentrations of organic acids and ethanol were found in sourdoughs back-slopped at relatively high temperatures (30 – 37°C) (Vrancken et al., 2011). Organic acids and FAA directly or indirectly affect the sensory aspects of baked goods (Cavallo et al., 2017; Pétel et al., 2017). One common complaint among bakers that use sourdough is the variability of technology performances, which in turn influences sensory traits of baked goods (Minervini et al., 2014). In this sense, sourdough microbial community behaves as that of another natural starter, namely natural whey starter culture used during manufacturing of some cheeses. For instance, *Lactobacillus delbrueckii* ssp. *bulgaricus* was found in natural whey starter until the 2000s (Bottazzi et al., 2000; Coppola et al., 2000), but later this species was no longer identified as a member of the microbiota of natural whey starter culture used for Parmigiano Reggiano PDO and Grana Padano PDO cheeses (Gatti et al., 2014). Variations were also found at the intraspecific level, depending on cheese and location of dairy plant (Gatti et al., 2014).

The results of this study highlight that strains of LAB vary during time in the same sourdough, even when applying the same flour and technology parameters. But what are the causes of this succession of strains? Most of the studies suggest that flour and house microbiota play key-roles in the stability/variability of sourdough microbiota (Gobbetti et al., 2016). In this study, house microbiota seemed to play a minor role, because its level of bacterial contamination was lower than that of flour. On the contrary, flour would seem to include two drivers of variability. The first is the inherent contamination of flour by LAB. Indeed, although the results from 16S metagenetic analysis indicated that flour bacterial microbiota was qualitatively constant, relative abundances of few genera and species varied during time, especially for samples from CG and MT. Although their cell density in flour is lower than in sourdough, flour autochthonous LAB may have the chance to become part of dominant bacterial biota of sourdough, as shown in this study and in previous papers (Siragusa et al., 2009; Minervini et al., 2010, 2018). This happens because flour is daily added to sourdough and at quite a high percentage (ca. 54 – 61% of dough weight in the case of the sourdoughs considered in this study). The second flour-inherent driver is the variability of nutrients contained. Fermentable carbohydrates and FAA in flour are influenced by endogenous amylases and proteases, respectively. Within the same type of flour, these enzyme activities may vary depending on the year (Muchová, 2003), harvesting season (Noda et al., 2003) and lapse of time between harvest and milling (Hrušková et al., 2004). Enzyme activities play a pivotal role in the sourdough microbial ecology (De Vuyst et al., 2009; Gänzle, 2014). As shown by permutation analysis, concentration of flour nutrients varied over 1 year and, in some cases, in a shorter time lapse (e.g., T5 vs. T6 for flours sampled in CG). It may be hypothesized that variations in nutrients may favor certain strains over others. Indeed, different fermentative profiles were found for sourdough isolates of *L. sanfranciscensis* (De Angelis et al., 2007). The same could occur for strains of *Weissella cibaria* and *Weissella confusa* (Fusco et al., 2015). A strain of *W. confusa* reached higher cell density when grown in MRS broth than in milk supplemented with yeast extract and glucose (Serna-Cock et al., 2012).

This study highlighted that flour was the main driver of stability of lactic acid bacterium biota in traditional sourdough, because it harbors contaminating bacterial strains and provides nutrients whose concentration varies during time. The evidences in this study lead to hypothesize that in order to make stable, at the strain level, a given traditional sourdough, an appropriate mixture of flours should be used so that their chemical composition (especially fermentable carbohydrates and FAA) does not vary during time. Constant chemical composition of flours mixture, together with the use of autochthonous intrinsically robust LAB strains, would be an advisable tool for overcoming the influence of microbial contamination, standardizing the fermentation process and, consequently, the quality of sourdough baked goods.

## Data Availability Statement

The raw data supporting the conclusions of this manuscript will be made available by the authors, without undue reservation, to any qualified researcher.

## Author Contributions

FM directed the experimental phases, elaborated and wrote the manuscript. FD performed the microbiological analyses, bio-typed and identified the bacterial strains. GC performed biochemical analyses on the flours and sourdoughs. MDA performed statistical analyses, discussed the results, and revised the manuscript. MG ideated the study, and revised the manuscript.

## Conflict of Interest Statement

The authors declare that the research was conducted in the absence of any commercial or financial relationships that could be construed as a potential conflict of interest.

## Abbreviations

AM: Altamura
CG: Castellana Grotte
LAB: lactic acid bacteria
mMRS: modified MRS
MT: Matera
RAPD: Randomly Amplified Polymorphic DNA
SDB: Sour Dough Bacteria.
