# Supplementary material for: Lactic Acid Bacterium Population Dynamics in Artisan Sourdoughs Over One Year of Daily Propagations Is Mainly Driven by Flour Microbiota and Nutrients
Source: Front Microbiol. 2018 Aug 27;9:1984. doi: 10.3389/fmicb.2018.01984 (PMC6119722; doi:10.3389/fmicb.2018.01984)
Supplement: Supplementary file 1 [file Table_1.DOCX]

Supplementary Material

**Lactic acid bacterium population dynamics in artisan sourdoughs over one year of daily propagations is mainly driven by flour microbiota and nutrients**

**Fabio Minervini, Francesca Rita Dinardo, Giuseppe Celano, Maria De Angelis, Marco Gobbetti***

*** Correspondence:** Marco Gobbetti: Marco.Gobbetti@unibz.it

**SUPPLEMENTARY TABLE 1.** Ingredients, temperature and time of incubation used for propagating the traditional sourdoughs object of study.

| Sourdoughs | Type of flour | Amount (g per kg of dough) | | |  |  |  |
| --- | --- | --- | --- | --- | --- | --- | --- |
|  |  | Flour | Sourdough | Water | Dough yield | T (°C) | time (h) |
| AM | *Triticum durum* | 614 | 110 | 276 | 145 | 20-26.5^*^ | 14 |
| CG | *Triticum aestivum* | 540 | 190 | 270 | 150 | 13-23^§^ | 4 - 7 |
| MT | *Triticum durum* | 625 | 62.5 | 312.5 | 152 | 10 | 24 |

^*^ Temperatures of sourdough fermentation were the following: T1, 22.5 °C; T2, 20 °C; T3, 20 °C; T4, 26.5 °C; T5, 23 °C; T6, 20 °C.

^§^ Temperatures of sourdough fermentation were the following: T1, 20 °C; T2, 13 °C; T3, 23 °C; T4, 20 °C; T5, 22 °C; T6, 20 °C .
