# Supplementary material for: Lactic Acid Bacterium Population Dynamics in Artisan Sourdoughs Over One Year of Daily Propagations Is Mainly Driven by Flour Microbiota and Nutrients
Source: Front Microbiol. 2018 Aug 27;9:1984. doi: 10.3389/fmicb.2018.01984 (PMC6119722; doi:10.3389/fmicb.2018.01984)
Supplement: Supplementary file 2 [file Table_2.DOCX]

***Supplementary Material***

**Lactic acid bacterium population dynamics in artisan sourdoughs over one year of daily propagations is mainly driven by flour microbiota and nutrients**

**Fabio Minervini, Francesca Rita Dinardo, Giuseppe Celano, Maria De Angelis, Marco Gobbetti***

*** Correspondence:** Marco Gobbetti: Marco.Gobbetti@unibz.it

**SUPPLEMENTARY TABLE 2.** Chemical characteristics of the tap waters used at the bakeries located in Altamura, Castellana Grotte and Matera.

|  | Fixed residue at 180°C (mg L^-1^) | Electric conductibility  (μS cm^-1^) | Bicarbonate  (mg L^-1^) | Ca^2+^  (mg L^-1^) | NO_4_^2-^  (mg L^-1^) | Fl^-^  (mg L^-1^) | Cl^-^  (mg L^-1^) | SO_4_^2-^  (mg L^-1^) | Na^+^  (mg L^-1^) | K^+^  (mg L^-1^) | Mg^2+^  (mg L^-1^) | pH | Hardness (mg of CaCO^3^ L^-1^) |
| --- | --- | --- | --- | --- | --- | --- | --- | --- | --- | --- | --- | --- | --- |
|  |  |  |  |  |  |  |  |  |  |  |  |  |  |
| Altamura^*^ | 230 | 328 | 201 | 53 | 2 | 0.14 | 12 | 21 | 11 | 1.8 | 11 | 7.90 | 170 |
| Castellana Grotte^*^ | 257 | 367 | 194 | 49 | 2 | 0.14 | 16 | 40 | 15 | 2.1 | 15 | 7.90 | 180 |
| Matera^¥^ | 257 | 368 | 204 | 58 | 2 | 0.10 | 14 | 26 | 11 | 2.0 | 12 | 7.88 | 190 |

* These data originate from the Acquedotto Pugliese website <http://www.aqp.it/portal/page/portal/MYAQP/SERVIZI/Qualita_Acqua>

^¥^ These data originate from the Acquedotto Lucano website https://www.acquedottolucano.it/le-analisi-dellacqua-del-tuo-comune/
