# Supplementary material for: Lactic Acid Bacterium Population Dynamics in Artisan Sourdoughs Over One Year of Daily Propagations Is Mainly Driven by Flour Microbiota and Nutrients
Source: Front Microbiol. 2018 Aug 27;9:1984. doi: 10.3389/fmicb.2018.01984 (PMC6119722; doi:10.3389/fmicb.2018.01984)
Supplement: Supplementary file 3 [file Table_3.DOCX]

Supplementary Material

**Lactic acid bacterium population dynamics in artisan sourdoughs over one year of daily propagations is mainly driven by flour microbiota and nutrients**

**Fabio Minervini, Francesca Rita Dinardo, Giuseppe Celano, Maria De Angelis, Marco Gobbetti***

*** Correspondence:** Marco Gobbetti: Marco.Gobbetti@unibz.it

**SUPPLEMENTARY TABLE 3** Culture media, method of inoculum, and temperature (°C) of incubation used for enumerating different microbial groups.

| **Microbial group** | **Agar medium for enumeration** | **Method of inoculum** | **Temp of incubation (°C)** |
| --- | --- | --- | --- |
| Lactic acid bacteria | Modified de Man-Rogosa-Sharpe (mMRS) with cycloheximide (0.1 g l^-1^) | Pour-plate | 30^a^ |
|  | Sour Dough Bacteria (SDB) with cycloheximide (0.1 g l^-1^) | Pour-plate | 30^a^ |
| Enterococci | Slanetz and Bartley | Spread-plate | 37 |
| Staphylococci and micrococci | Baird Parker | Spread-plate | 30 |
| Total coliforms | Violet Red Bile Glucose | Pour-plate | 37 |
| Acetic acid bacteria | Deoxycholate-mannitol-sorbitol | Pour-plate | 30 |
| Molds | Wort agar | Pour-plate | 30 |
| Yeasts | Sabouraud Dextrose with chloramphenicol (0.1 g l^-1^) | Pour-plate | 30 |

^a^ under anaerobic conditions (AnaeroGen, Oxoid)
