# Supplementary material for: Lactic Acid Bacterium Population Dynamics in Artisan Sourdoughs Over One Year of Daily Propagations Is Mainly Driven by Flour Microbiota and Nutrients
Source: Front Microbiol. 2018 Aug 27;9:1984. doi: 10.3389/fmicb.2018.01984 (PMC6119722; doi:10.3389/fmicb.2018.01984)
Supplement: Supplementary file 4 [file Table_4.DOCX]

Supplementary Material

**Lactic acid bacterium population dynamics in artisan sourdoughs over one year of daily propagations is mainly driven by flour microbiota and nutrients**

**Fabio Minervini, Francesca Rita Dinardo, Giuseppe Celano, Maria De Angelis, Marco Gobbetti***

*** Correspondence:** Marco Gobbetti: Marco.Gobbetti@unibz.it

**SUPPLEMENTARY TABLE 4** Cell numbers^a^ (log cfu/g or log cfu/cm^2^) of sub-dominant microbial groups in the flours (F), sourdoughs (S), and bakery environment (surfaces of dough mixer, DM, and storage box, SB) sampled in Altamura, Castellana Grotte and Matera every two months.

| **Sampling time and sample code** | **Staphylococci and micrococci** | **Coliforms** | **Acetic acid bacteria** | **Molds** |
| --- | --- | --- | --- | --- |
|  | **Altamura** | | | |
| T1-F | 3.5±0.4 | 4.0±0.3 | <1 | 4.0±0.0 |
| T1-S | 4.2±0.8 | 1.8±0.7 | <1 | <1 |
| T1-DM | 0.9±0.2 | 1.5±1.4 | <0.025 | 2.8±0.0 |
| T1 SB | 1.5±0.3 | 2.0±0.5 | <0.025 | 3.0±0.1 |
| T2-F | 2.8±0.0 | 4.3±0.2 | <1 | 3.8±0.7 |
| T2-S | 3.7±0.1 | <1 | <1 | <1 |
| T2-DM | <0.25 | <0.025 | <0.025 | <0.025 |
| T2 SB | <0.25 | 0.1±0.0 | <0.025 | 1.8±0.0 |
| T3-F | <2 | 4.2±0.0 | <1 | 3.8±0.0 |
| T3-S | 5.1±0.5 | <1 | 5.4±1.1 | 4.6±0.0 |
| T3-DM | 2.1±0.0 | 1.4±0.0 | <0.025 | <0.025 |
| T3 SB | <0.25 | 1.4±0.0 | <0.025 | 0.6±0.0 |
| T4-F | 4.4±0.3 | 3.7±0.3 | <1 | <1 |
| T4-S | 4.1±0.4 | 1.7±0.0 | 5.9±1.1 | <1 |
| T4-DM | <0.25 | <0.025 | 1.4±0.0 | <0.025 |
| T4 SB | <0.25 | <0.025 | 1.5±0.0 | 1.3±0.0 |
| T5-F | 3.7±0.5 | 3.3±0.4 | <1 | <1 |
| T5-S | <2 | 1.1±0.2 | 6.0±0.4 | <1 |
| T5-DM | <0.25 | <0.025 | <0.025 | 2.0±0.7 |
| T5 SB | <0.25 | <0.025 | 1.7±0.4 | 2.3±0.0 |
| T6-F | 2.5±0.3 | 4.1±0.0 | <1 | <1 |
| T6-S | 5.1±0.2 | 2.4±0.0 | 5.2±0.2 | <1 |
| T6-DM | <0.25 | <0.025 | <0.025 | <0.025 |
| T6 SB | 0.9±0.4 | <0.025 | <0.025 | <0.025 |
|  | **Castellana Grotte** | | | |
| T1-F | 3.5±0.6 | 4.0±1.0 | <1 | 3.0±0.0 |
| T1-S | 4.5±0.3 | 1.6±0.3 | 2.9±0.0 | <1 |
| T1-DM | 1.2±0.8 | <0.025 | <0.025 | 1.1±0.0 |
| T1 SB | 2.8±0.1 | <0.025 | <0.025 | 1.6±0.2 |
| T2-F | 2.0±1.7 | 2.5±0.6 | <1 | 2.6±0.7 |
| T2-S | 3.9±1.8 | 2.1±0.9 | <1 | 1.0±0.1 |
| T2-DM | <0.25 | <0.025 | <0.025 | <0.025 |
| T2 SB | <0.25 | <0.025 | <0.025 | <0.025 |
| T3-F | 3.4±0.2 | 4.7±1.6 | <1 | <1 |
| T3-S | 3.6±0.1 | <1 | 4.4±1.3 | <1 |
| T3-DM | <0.25 | 3.5±0.0 | <0.025 | <0.025 |
| T3 SB | <0.25 | 1.3±0.8 | 2.5±0.0 | 1.0±0.4 |
| T4-F | 3.5±0.6 | 2.0±0.0 | <1 | 3.9±0.0 |
| T4-S | 3.5±0.6 | 2.5±0.3 | 3.4±0.2 | 1.0±0.0 |
| T4-DM | <0.25 | <0.025 | <0.025 | <0.025 |
| T4 SB | 2.6±0.5 | <0.025 | <0.025 | 2.0±0.6 |
| T5-F | 3.3±0.3 | 3.3±0.0 | <1 | <1 |
| T5-S | 3.7±0.4 | 2.4±1.2 | 3.8±0.1 | 1.4±0.3 |
| T5-DM | <0.25 | <0.025 | <0.025 | <0.025 |
| T5 SB | 0.3±0.0 | <0.025 | <0.025 | <0.025 |
| T6-F | 2.5±0.3 | 2.0±0.0 | <1 | <1 |
| T6-S | 5.2±0.2 | 1.8±0.7 | 3.4±0.2 | <1 |
| T6-DM | 0.6±0.0 | <0.025 | <0.025 | 1.1±0.0 |
| T6 SB | 0.9±0.4 | <0.025 | <0.025 | <0.025 |
|  | **Matera** | | | |
| T1-F | 3.8±0.2 | 3.6±0.5 | <1 | 3.3±0.0 |
| T1-S | 4.6±0.5 | 3.3±0.4 | <1 | 2.4±0.0 |
| T1-DM | <0.25 | 1.8±0.0 | <0.025 | <0.025 |
| T1 SB | 1.8±0.0 | 0.1±0.0 | <0.025 | <0.025 |
| T2-F | 3.1±0.2 | 5.1±0.7 | <1 | 4.2±0.9 |
| T2-S | 2.7±0.0 | 3.4±0.2 | <1 | 3.5±0.0 |
| T2-DM | 0.3±0.0 | <0.025 | <0.025 | 0.1±0.0 |
| T2 SB | 1.4±0.0 | <0.025 | <0.025 | 1.1±0.0 |
| T3-F | 3.1±0.2 | 4.9±1.1 | <1 | 4.4±0.0 |
| T3-S | 4.3±0.4 | 4.3±0.6 | <1 | 3.5±0.0 |
| T3-DM | <0.25 | 3.3±0.0 | <0.025 | <0.025 |
| T3 SB | 2.5±0.8 | 3.2±0.0 | 1.6±0.0 | 2.7±0.8 |
| T4-F | 3.6±0.4 | 4.5±0.2 | 1.4±0.9 | 2.8±0.0 |
| T4-S | 4.2±0.6 | 3.1±0.4 | 1.6±0.5 | 3.5±0.0 |
| T4-DM | 1.3±0.5 | 2.3±0.0 | 1.0±0.5 | <0.025 |
| T4 SB | 2.1±0.0 | <0.025 | 0.5±0.3 | 0.4±0.1 |
| T5-F | 3.1±0.2 | 3.6±0.7 | <1 | <1 |
| T5-S | 4.5±0.5 | 3.5±0.9 | 1.2±0.5 | <1 |
| T5-DM | <0.25 | <0.025 | <0.025 | <0.025 |
| T5 SB | 0.6±0.0 | <0.025 | <0.025 | <0.025 |
| T6-F | 3.3±0.1 | 5.4±0.0 | <1 | 4.4±0.0 |
| T6-S | 4.7±0.5 | 3.6±0.9 | 2.6±0.0 | 1.8±0.0 |
| T6-DM | <0.25 | <0.025 | 0.8±0.0 | <0.025 |
| T6 SB | 0.6±0.5 | <0.025 | <0.025 | <0.025 |

^a^ Mean values of three replicates
