# Supplementary material for: Lactic Acid Bacterium Population Dynamics in Artisan Sourdoughs Over One Year of Daily Propagations Is Mainly Driven by Flour Microbiota and Nutrients
Source: Front Microbiol. 2018 Aug 27;9:1984. doi: 10.3389/fmicb.2018.01984 (PMC6119722; doi:10.3389/fmicb.2018.01984)
Supplement: Supplementary file 5 [file Table_5.DOCX]

Supplementary Material

**Lactic acid bacterium population dynamics in artisan sourdoughs over one year of daily propagations is mainly driven by flour microbiota and nutrients**

**Fabio Minervini, Francesca Rita Dinardo, Giuseppe Celano, Maria De Angelis, Marco Gobbetti***

*** Correspondence:** Marco Gobbetti: Marco.Gobbetti@unibz.it

**SUPPLEMENTARY TABLE 5** Relative abundance (%)^a^ of bacterial OTUs classified at the highest possible taxonomic level (species/genus/family) found in the flour (F) and sourdough (S) sampled at Altamura bakery at month 1 (T1), 6 (T3) and 12 (T6). Only OTUs with a relative abundance ≥ 0.1% in at least one sample are shown.

|  | T1 | | T3 | T6 | |
| --- | --- | --- | --- | --- | --- |
| Taxon | F | S | S | F | S |
| *Bacillus* | 0.260 | 0.000 | 0.000 | 0.083 | 0.000 |
| *Lactobacillus sanfranciscensis* | 24.826 | 99.914 | 98.403 | 24.358 | 98.991 |
| *Weissella confusa/cibaria* | 0.478 | 0.008 | 0.259 | 0.392 | 0.195 |
| *Leuconostoc* | 0.069 | 0.000 | 1.234 | 0.027 | 0.137 |
| *Enterobacteriaceae* | 2.198 | 0.004 | 0.000 | 2.000 | 0.048 |
| *Erwinia* | 6.907 | 0.005 | 0.000 | 6.009 | 0.109 |
| *Pantoea agglomerans* | 0.256 | 0.002 | 0.000 | 0.243 | 0.009 |
| *Acinetobacter* | 0.423 | 0.001 | 0.000 | 0.151 | 0.009 |
| *Acinetobacter johnsonii* | 0.721 | 0.000 | 0.000 | 0.260 | 0.093 |
| *Pseudomonadaceae* | 0.679 | 0.000 | 0.000 | 0.801 | 0.011 |
| *Pseudomonas* | 61.053 | 0.030 | 0.000 | 63.958 | 0.301 |
| Others | 2.130 | 0.035 | 0.104 | 1.718 | 0.096 |

^a^ Mean values of three replicates
