# Supplementary material for: Lactic Acid Bacterium Population Dynamics in Artisan Sourdoughs Over One Year of Daily Propagations Is Mainly Driven by Flour Microbiota and Nutrients
Source: Front Microbiol. 2018 Aug 27;9:1984. doi: 10.3389/fmicb.2018.01984 (PMC6119722; doi:10.3389/fmicb.2018.01984)
Supplement: Supplementary file 6 [file Table_6.DOCX]

***Supplementary Material***

**Lactic acid bacterium population dynamics in artisan sourdoughs over one year of daily propagations is mainly driven by flour microbiota and nutrients**

**Fabio Minervini, Francesca Rita Dinardo, Giuseppe Celano, Maria De Angelis, Marco Gobbetti***

*** Correspondence:** Marco Gobbetti: Marco.Gobbetti@unibz.it

**SUPPLEMENTARY TABLE 6.** Relative abundance (%)^a^ of bacterial OTUs classified at the highest possible taxonomic level (species/genus/family) found in the flour (F) sourdough (S), and environment (E) sampled at Castellana Grotte bakery at month 1 (T1), 6 (T3) and 12 (T6). Only OTUs with a relative abundance ≥ 1% in at least one sample are shown.

|  | T1 | | | T3 | T6 | | |
| --- | --- | --- | --- | --- | --- | --- | --- |
| Taxon | F | S | E | S | F | S | E |
| *Bifidobacterium adolescentis* | 0.000 | 0.000 | 1.164 | 0.000 | 0.000 | 0.000 | 0.443 |
| *Bifidobacterium longum* | 0.000 | 0.000 | 1.963 | 0.000 | 0.000 | 0.000 | 0.636 |
| *Coriobacteriaceae* | 0.000 | 0.000 | 1.286 | 0.000 | 0.000 | 0.000 | 0.549 |
| *Collinsella aerofaciens* | 0.000 | 0.000 | 4.661 | 0.000 | 0.000 | 0.000 | 1.464 |
| *Bacteroides* | 0.002 | 0.003 | 15.241 | 0.309 | 0.003 | 0.001 | 6.994 |
| *Bacteroides uniformis* | 0.000 | 0.000 | 4.866 | 0.000 | 0.000 | 0.000 | 2.244 |
| *Prevotella* | 0.000 | 0.000 | 4.734 | 0.000 | 0.000 | 0.000 | 1.869 |
| *Paenibacillus* | 0.000 | 0.000 | 0.011 | 0.000 | 0.003 | 0.001 | 1.740 |
| *Staphylococcus* | 0.026 | 0.000 | 0.016 | 0.000 | 0.176 | 0.001 | 1.952 |
| *Lactobacillus sakei* | 1.879 | 0.006 | 0.020 | 0.000 | 0.094 | 1.821 | 1.947 |
| *Lactobacillus sanfranciscensis* | 50.968 | 99.626 | 4.829 | 98.900 | 2.581 | 98.033 | 41.286 |
| *Leuconostoc* | 1.456 | 0.001 | 0.038 | 0.000 | 0.246 | 0.001 | 1.820 |
| *Clostridiales* | 0.000 | 0.000 | 4.651 | 0.000 | 0.001 | 0.000 | 2.179 |
| *Clostridiaceae* | 0.000 | 0.000 | 1.168 | 0.000 | 0.000 | 0.000 | 0.569 |
| *Lachnospiraceae* | 0.002 | 0.001 | 7.120 | 0.000 | 0.001 | 0.000 | 3.343 |
| *Anaerostipes* | 0.000 | 0.000 | 2.725 | 0.000 | 0.001 | 0.000 | 1.342 |
| *Blautia* | 0.000 | 0.001 | 2.100 | 0.000 | 0.000 | 0.001 | 1.162 |
| *Roseburia* | 0.000 | 0.000 | 1.328 | 0.000 | 0.002 | 0.000 | 0.569 |
| *Ruminococcaceae* | 0.001 | 0.000 | 8.793 | 0.000 | 0.002 | 0.000 | 4.763 |
| *Faecalibacterium prausnitzii* | 0.001 | 0.000 | 9.407 | 0.000 | 0.000 | 0.000 | 5.080 |
| *Ruminococcus* | 0.001 | 0.001 | 6.189 | 0.000 | 0.001 | 0.000 | 2.989 |
| *Dialister* | 0.000 | 0.000 | 3.624 | 0.000 | 0.000 | 0.001 | 2.055 |
| *Erysipelotrichaceae* | 0.000 | 0.000 | 1.622 | 0.000 | 0.000 | 0.000 | 0.594 |
| *Sutterella* | 0.000 | 0.000 | 2.469 | 0.000 | 0.000 | 0.000 | 0.868 |
| *Erwinia* | 3.680 | 0.023 | 0.025 | 0.000 | 5.076 | 0.003 | 0.083 |
| *Pseudomonas* | 38.885 | 0.211 | 0.010 | 0.343 | 85.848 | 0.093 | 0.021 |
| Others | 3.099 | 0.128 | 9.941 | 0.447 | 5.967 | 0.045 | 11.439 |

^a^ Mean values of three replicates
