# Supplementary material for: Lactic Acid Bacterium Population Dynamics in Artisan Sourdoughs Over One Year of Daily Propagations Is Mainly Driven by Flour Microbiota and Nutrients
Source: Front Microbiol. 2018 Aug 27;9:1984. doi: 10.3389/fmicb.2018.01984 (PMC6119722; doi:10.3389/fmicb.2018.01984)
Supplement: Supplementary file 7 [file Table_7.DOCX]

***Supplementary Material***

**Lactic acid bacterium population dynamics in artisan sourdoughs over one year of daily propagations is mainly driven by flour microbiota and nutrients**

**Fabio Minervini, Francesca Rita Dinardo, Giuseppe Celano, Maria De Angelis, Marco Gobbetti***

*** Correspondence:** Marco Gobbetti: Marco.Gobbetti@unibz.it

**SUPPLEMENTARY TABLE 7.** Relative abundance (%)^a^ of bacterial OTUs classified at the highest possible taxonomic level (species/genus/family) found in the flour (F) and sourdough (S) sampled at Matera bakery at month 1 (T1), 6 (T3) and 12 (T6). Only OTUs with a relative abundance ≥ 0.1% in at least one sample are shown.

|  | T1 | | T3 | T6 | |
| --- | --- | --- | --- | --- | --- |
| Taxon | F | S | S | F | S |
| *Lactobacillus sanfranciscensis* | 5.916 | 0.012 | 2.832 | 2.595 | 0.182 |
| *Pediococcus* | 0.102 | 0.003 | 0.000 | 0.542 | 0.001 |
| *Weissella confusa/cibaria* | 2.807 | 97.813 | 94.308 | 1.744 | 97.540 |
| *Leuconostoc* | 6.298 | 0.000 | 0.000 | 0.041 | 0.000 |
| *Weissella cibaria* | 0.060 | 1.975 | 0.566 | 0.039 | 2.006 |
| *Sphingomonadaceae* | 0.266 | 0.000 | 0.000 | 0.004 | 0.001 |
| *Enterobacteriaceae* | 2.266 | 0.001 | 0.399 | 2.248 | 0.003 |
| *Erwinia* | 7.635 | 0.003 | 0.228 | 7.297 | 0.006 |
| *Pantoea agglomerans* | 0.277 | 0.000 | 0.000 | 0.323 | 0.000 |
| *Acinetobacter johnsonii* | 0.332 | 0.005 | 1.160 | 0.243 | 0.029 |
| *Enhydrobacter* | 0.630 | 0.000 | 0.000 | 0.025 | 0.004 |
| *Pseudomonadaceae* | 0.769 | 0.002 | 0.000 | 1.018 | 0.002 |
| *Pseudomonas* | 70.642 | 0.150 | 0.387 | 81.990 | 0.136 |
| Others | 2.000 | 0.035 | 0.122 | 1.891 | 0.090 |

^a^ Mean values of three replicates
