# Supplementary material for: Lactic Acid Bacterium Population Dynamics in Artisan Sourdoughs Over One Year of Daily Propagations Is Mainly Driven by Flour Microbiota and Nutrients
Source: Front Microbiol. 2018 Aug 27;9:1984. doi: 10.3389/fmicb.2018.01984 (PMC6119722; doi:10.3389/fmicb.2018.01984)
Supplement: Supplementary file 8 [file Table_8.DOCX]

Supplementary Material

**Lactic acid bacterium population dynamics in artisan sourdoughs over one year of daily propagations is mainly driven by flour microbiota and nutrients**

**Fabio Minervini, Francesca Rita Dinardo, Giuseppe Celano, Maria De Angelis, Marco Gobbetti***

*** Correspondence:** Marco Gobbetti: Marco.Gobbetti@unibz.it

**SUPPLEMENTARY TABLE 8.** Concentrations^a^ of fermentable carbohydrates (in mM), total and individual free amino acids (FAA) (in mg kg^-1^) in flours used for back slopping of sourdoughs sampled in Altamura, Castellana Grotte and Matera every two months.

|  | **Altamura** | | | | | | **Castellana Grotte** | | | | | | **Matera** | | | | | |
| --- | --- | --- | --- | --- | --- | --- | --- | --- | --- | --- | --- | --- | --- | --- | --- | --- | --- | --- |
|  | **T1-F** | **T2-F** | **T3-F** | **T4-F** | **T5-F** | **T6-F** | **T1-F** | **T2-F** | **T3-F** | **T4-F** | **T5-F** | **T6-F** | **T1-F** | **T2-F** | **T3-F** | **T4-F** | **T5-F** | **T6-F** |
| Glucose | 8 | 7 | 7 | 7 | 7 | 7 | 14 | 8 | 8 | 8 | 18 | 15 | 6 | 7 | 9 | 9 | 6 | 6 |
| Fructose | 7 | 7 | 6 | 7 | 8 | 8 | 13 | 8 | 6 | 9 | 21 | 16 | 7 | 7 | 8 | 8 | 8 | 9 |
| Sucrose | 13 | 8 | 7 | 6 | 8 | 5 | 3 | 2 | 5 | 3 | 3 | 1 | 9 | 7 | 7 | 12 | 11 | 7 |
| Maltose | 46 | 29 | 30 | 34 | 11 | 23 | 24 | 23 | 16 | 14 | 8 | 31 | 25 | 43 | 23 | 44 | 31 | 42 |
| Total FAA | 534 | 513 | 626 | 558 | 497 | 712 | 577 | 540 | 475 | 582 | 519 | 504 | 581 | 788 | 605 | 623 | 523 | 972 |
| Asp | 165 | 170 | 199 | 184 | 105 | 233 | 161 | 168 | 148 | 185 | 107 | 162 | 169 | 210 | 173 | 179 | 89 | 251 |
| Thr | 6 | 0 | 0 | 0 | 0 | 0 | 0 | 0 | 6 | 7 | 0 | 0 | 0 | 0 | 0 | 0 | 0 | 0 |
| Ser | 8 | 6 | 38 | 22 | 62 | 38 | 40 | 34 | 17 | 31 | 78 | 34 | 35 | 115 | 50 | 48 | 77 | 137 |
| Glu | 100 | 96 | 108 | 97 | 74 | 110 | 107 | 99 | 101 | 106 | 92 | 89 | 96 | 149 | 118 | 123 | 82 | 170 |
| Gly | 16 | 14 | 18 | 17 | 11 | 18 | 15 | 14 | 11 | 14 | 13 | 11 | 16 | 21 | 18 | 20 | 12 | 26 |
| Ala | 23 | 19 | 22 | 19 | 23 | 29 | 32 | 27 | 19 | 30 | 31 | 25 | 23 | 38 | 22 | 25 | 26 | 44 |
| Cys | 26 | 25 | 23 | 24 | 15 | 22 | 22 | 18 | 16 | 16 | 11 | 17 | 25 | 25 | 24 | 25 | 16 | 25 |
| Val | 16 | 16 | 18 | 18 | 14 | 19 | 17 | 19 | 19 | 19 | 17 | 20 | 17 | 22 | 20 | 18 | 15 | 30 |
| Met | 0 | 0 | 0 | 0 | 5 | 2 | 2 | 3 | 2 | 4 | 8 | 6 | 0 | 3 | 0 | 0 | 5 | 3 |
| Ile | 4 | 4 | 4 | 4 | 6 | 6 | 6 | 7 | 5 | 7 | 9 | 6 | 4 | 5 | 5 | 4 | 7 | 6 |
| Leu | 7 | 12 | 12 | 11 | 8 | 15 | 9 | 11 | 11 | 10 | 11 | 9 | 12 | 12 | 11 | 9 | 9 | 12 |
| Tyr | 18 | 23 | 22 | 25 | 21 | 13 | 12 | 7 | 8 | 8 | 14 | 6 | 14 | 16 | 17 | 19 | 21 | 17 |
| Phe | 9 | 11 | 10 | 12 | 6 | 16 | 14 | 11 | 9 | 10 | 7 | 9 | 13 | 11 | 12 | 12 | 8 | 12 |
| GABA | 11 | 14 | 9 | 9 | 13 | 8 | 19 | 21 | 15 | 12 | 15 | 12 | 10 | 16 | 10 | 7 | 15 | 11 |
| His | 5 | 0 | 7 | 2 | 6 | 2 | 7 | 4 | 5 | 0 | 7 | 0 | 6 | 1 | 1 | 1 | 7 | 60 |
| Trp | 66 | 74 | 84 | 82 | 61 | 122 | 62 | 56 | 52 | 61 | 34 | 46 | 78 | 62 | 74 | 86 | 67 | 92 |
| Orn | 4 | 4 | 0 | 0 | 7 | 3 | 3 | 2 | 0 | 0 | 6 | 0 | 4 | 5 | 0 | 0 | 5 | 2 |
| Lys | 11 | 8 | 11 | 9 | 20 | 11 | 11 | 12 | 9 | 12 | 15 | 11 | 12 | 16 | 13 | 13 | 20 | 19 |
| Arg | 12 | 4 | 7 | 7 | 14 | 9 | 18 | 14 | 9 | 19 | 20 | 16 | 12 | 15 | 18 | 15 | 16 | 11 |
| Pro | 28 | 14 | 34 | 17 | 25 | 36 | 18 | 12 | 14 | 33 | 23 | 26 | 33 | 44 | 21 | 19 | 25 | 44 |

^a^ Mean values of three replicates
