# Supplementary material for: Lactic Acid Bacterium Population Dynamics in Artisan Sourdoughs Over One Year of Daily Propagations Is Mainly Driven by Flour Microbiota and Nutrients
Source: Front Microbiol. 2018 Aug 27;9:1984. doi: 10.3389/fmicb.2018.01984 (PMC6119722; doi:10.3389/fmicb.2018.01984)
Supplement: Supplementary file 9 [file Table_9.DOCX]

Supplementary Material

**Lactic acid bacterium population dynamics in artisan sourdoughs over one year of daily propagations is mainly driven by flour microbiota and nutrients**

**Fabio Minervini, Francesca Rita Dinardo, Giuseppe Celano, Maria De Angelis, Marco Gobbetti***

*** Correspondence:** Marco Gobbetti: Marco.Gobbetti@unibz.it

**SUPPLEMENTARY TABLE 9.** pH and concentrations^a^ of residual fermentable carbohydrates, lactic acid, acetic acid, ethanol (in mM), total and individual free amino acids (FAA) (in mg kg^-1^) in sourdoughs sampled in Altamura, Castellana Grotte and Matera every two months.

|  | **Altamura** | | | | | | **Castellana Grotte** | | | | | | **Matera** | | | | | |
| --- | --- | --- | --- | --- | --- | --- | --- | --- | --- | --- | --- | --- | --- | --- | --- | --- | --- | --- |
|  | **T1-S** | **T2-S** | **T3-S** | **T4-S** | **T5-S** | **T6-S** | **T1-S** | **T2-S** | **T3-S** | **T4-S** | **T5-S** | **T6-S** | **T1-S** | **T2-S** | **T3-S** | **T4-S** | **T5-S** | **T6-S** |
| pH | 4.13 | 4.13 | 4.00 | 4.10 | 3.75 | 3.80 | 3.90 | 4.30 | 4.37 | 4.47 | 4.57 | 4.53 | 4.47 | 4.63 | 4.80 | 4.67 | 4.73 | 4.63 |
| Glucose | 20 | 6 | 11 | 7 | 15 | 16 | 15 | 10 | 9 | 13 | 26 | 21 | 19 | 9 | 29 | 10 | 12 | 13 |
| Fructose | 7 | 3 | 5 | 2 | 7 | 3 | 11 | 7 | 7 | 8 | 10 | 12 | 6 | 2 | 6 | 5 | 4 | 13 |
| Sucrose | 0 | 0 | 0 | 1 | 0 | 0 | 1 | 0 | 0 | 1 | 0 | 1 | 0 | 0 | 0 | 0 | 6 | 1 |
| Maltose | 9 | 14 | 10 | 14 | 2 | 1 | 15 | 12 | 17 | 14 | 12 | 12 | 10 | 25 | 30 | 25 | 20 | 18 |
| Lactic acid | 50 | 56 | 64 | 60 | 74 | 74 | 53 | 42 | 42 | 40 | 39 | 40 | 40 | 35 | 32 | 35 | 32 | 40 |
| Acetic acid | 10 | 13 | 12 | 14 | 11 | 11 | 10 | 13 | 12 | 9 | 7 | 5 | 20 | 15 | 12 | 8 | 11 | 7 |
| Ethanol | 96 | 88 | 89 | 98 | 91 | 87 | 101 | 104 | 100 | 93 | 82 | 70 | 97 | 106 | 110 | 104 | 103 | 107 |
| Total FAA | 1298 | 958 | 1022 | 686 | 2274 | 2230 | 4260 | 1139 | 980 | 1135 | 2645 | 2903 | 3743 | 1859 | 972 | 955 | 951 | 1362 |
| Asp | 39 | 47 | 146 | 21 | 231 | 261 | 153 | 61 | 108 | 209 | 423 | 71 | 23 | 74 | 64 | 50 | 62 | 105 |
| Thr | 47 | 7 | 23 | 8 | 51 | 87 | 124 | 43 | 9 | 13 | 62 | 62 | 58 | 108 | 31 | 38 | 40 | 27 |
| Ser | 74 | 26 | 17 | 30 | 76 | 150 | 234 | 72 | 43 | 28 | 119 | 92 | 256 | 133 | 34 | 43 | 54 | 102 |
| Glu | 145 | 105 | 99 | 80 | 247 | 406 | 1261 | 175 | 98 | 131 | 306 | 1370 | 1214 | 246 | 117 | 116 | 98 | 166 |
| Gly | 33 | 32 | 39 | 30 | 86 | 70 | 151 | 28 | 41 | 27 | 75 | 84 | 180 | 79 | 37 | 35 | 38 | 46 |
| Ala | 60 | 49 | 72 | 44 | 217 | 169 | 256 | 70 | 42 | 42 | 144 | 145 | 243 | 149 | 72 | 77 | 67 | 83 |
| Cys | 43 | 37 | 37 | 25 | 50 | 44 | 45 | 34 | 19 | 37 | 45 | 40 | 59 | 46 | 45 | 47 | 40 | 34 |
| Val | 40 | 51 | 48 | 31 | 126 | 100 | 214 | 73 | 43 | 46 | 140 | 95 | 224 | 92 | 52 | 53 | 56 | 46 |
| Met | 35 | 21 | 13 | 14 | 61 | 48 | 66 | 19 | 19 | 11 | 63 | 19 | 59 | 33 | 14 | 15 | 22 | 14 |
| Ile | 14 | 17 | 18 | 12 | 77 | 43 | 128 | 23 | 23 | 25 | 107 | 72 | 165 | 37 | 21 | 25 | 35 | 20 |
| Leu | 18 | 51 | 74 | 40 | 202 | 69 | 275 | 49 | 64 | 69 | 248 | 127 | 255 | 45 | 42 | 50 | 70 | 43 |
| Tyr | 65 | 48 | 31 | 29 | 69 | 73 | 140 | 0 | 42 | 27 | 80 | 90 | 140 | 77 | 16 | 20 | 31 | 52 |
| Phe | 82 | 32 | 38 | 22 | 108 | 131 | 341 | 79 | 51 | 49 | 134 | 221 | 269 | 84 | 23 | 26 | 36 | 30 |
| GABA | 13 | 76 | 63 | 58 | 113 | 2 | 21 | 6 | 74 | 64 | 163 | 36 | 90 | 0 | 44 | 43 | 26 | 107 |
| His | 150 | 15 | 8 | 5 | 4 | 31 | 4 | 141 | 20 | 138 | 4 | 17 | 23 | 27 | 2 | 11 | 11 | 23 |
| Trp | 118 | 103 | 92 | 90 | 148 | 204 | 141 | 0 | 64 | 84 | 105 | 37 | 57 | 119 | 55 | 48 | 28 | 88 |
| Orn | 10 | 25 | 0 | 9 | 0 | 11 | 83 | 5 | 13 | 0 | 0 | 56 | 134 | 19 | 19 | 4 | 4 | 37 |
| Lys | 125 | 50 | 22 | 35 | 87 | 138 | 187 | 99 | 66 | 30 | 75 | 5 | 19 | 211 | 88 | 102 | 92 | 130 |
| Arg | 83 | 62 | 95 | 26 | 213 | 89 | 154 | 49 | 74 | 73 | 252 | 0 | 0 | 119 | 88 | 108 | 98 | 83 |
| Pro | 104 | 104 | 86 | 77 | 107 | 104 | 281 | 115 | 67 | 32 | 100 | 266 | 274 | 160 | 107 | 48 | 42 | 125 |

^a^ Mean values of three replicates
