# Supplementary material for: Lactic Acid Bacterium Population Dynamics in Artisan Sourdoughs Over One Year of Daily Propagations Is Mainly Driven by Flour Microbiota and Nutrients
Source: Front Microbiol. 2018 Aug 27;9:1984. doi: 10.3389/fmicb.2018.01984 (PMC6119722; doi:10.3389/fmicb.2018.01984)
Supplement: Supplementary file 10 [file Table_10.DOCX]

Supplementary Material

**Lactic acid bacterium population dynamics in artisan sourdoughs over one year of daily propagations is mainly driven by flour microbiota and nutrients**

**Fabio Minervini, Francesca Rita Dinardo, Giuseppe Celano, Maria De Angelis, Marco Gobbetti***

*** Correspondence:** Marco Gobbetti: Marco.Gobbetti@unibz.it

**SUPPLEMENTARY TABLE 10** Correlations between fermentation temperature and pH, and concentrations of lactic acid and maltose of sourdoughs of sourdoughs collected at the three bakeries at different sampling points.

| Sampling point |  | pH | lactic acid | maltose |
| --- | --- | --- | --- | --- |
| T1 | temperature | -0.82^*^ | 0.92^*^ | 0.18 |
|  | pH | - | -0.98^*^ | -0.70^*^ |
| T2 | temperature | -0.92^*^ | 0.99^*^ | -0.63 |
|  | pH | - | -0.93^*^ | 0.88^*^ |
| T3 | temperature | -0.76^*^ | 0.57 | -0.84^*^ |
|  | pH | - | -0.97^*^ | 0.99^*^ |
| T4 | temperature | -0.96^*^ | 0.90^*^ | -0.92^*^ |
|  | pH | - | -0.99^*^ | 0.77^*^ |
| T5 | temperature | -0.68 | 0.68 | -0.87^*^ |
|  | pH | - | -0.99^*^ | 0.95^*^ |
| T6 | temperature | -0.59 | 0.50 | -0.77^*^ |
|  | pH | - | -0.99^*^ | 0.97^*^ |

^*^ Significant correlations found at p< 0.05.
