# Supplementary material for: Lactic Acid Bacterium Population Dynamics in Artisan Sourdoughs Over One Year of Daily Propagations Is Mainly Driven by Flour Microbiota and Nutrients
Source: Front Microbiol. 2018 Aug 27;9:1984. doi: 10.3389/fmicb.2018.01984 (PMC6119722; doi:10.3389/fmicb.2018.01984)
Supplement: Supplementary file 11 [file Table_11.DOCX]

Supplementary Material

**Lactic acid bacterium population dynamics in artisan sourdoughs over one year of daily propagations is mainly driven by flour microbiota and nutrients**

**Fabio Minervini, Francesca Rita Dinardo, Giuseppe Celano, Maria De Angelis, Marco Gobbetti***

*** Correspondence:** Marco Gobbetti: Marco.Gobbetti@unibz.it

**SUPPLEMENTARY TABLE 11** Correlations between LAB cell density (on mMRS and SDB), pH, concentrations of lactic acid, acetic acid, ethanol, glucose, fructose, sucrose, maltose, FAA in the sourdoughs sampled at AM and CG bakeries and fermentation temperature and sourdough pH recorded at the same bakeries during the monitoring campaign.

|  | AM | | CG | |
| --- | --- | --- | --- | --- |
|  | temperature | pH | temperature | pH |
| LAB cell density on mMRS | -0.34 | -0.92^*^ | 0.53 | -0.45 |
| LAB cell density on SDB | 0.03 | -0.88^*^ | -0.45 | -0.91^*^ |
| Lactic acid | -0.15 | -0.95^*^ | -0.05 | -0.98^*^ |
| Acetic acid | 0.37 | 0.40 | -0.42 | -0.48 |
| Ethanol | 0.89^*^ | 0.47 | -0.35 | -0.65 |
| pH | 0.18 | - | 0.20 | - |
| Glucose | -0.13 | -0.37 | 0.35 | 0.41 |
| Fructose | -0.09 | -0.24 | 0.28 | -0.08 |
| Sucrose | 0.85^*^ | 0.33 | 0.10 | -0.25 |
| Maltose | 0.27 | 0.92^*^ | 0.51 | -0.43 |
| FAA | -0.27 | -0.91^*^ | 0.20 | -0.50 |

^*^Significant correlations found at p< 0.05.
