# Supplementary material for: Lactic Acid Bacterium Population Dynamics in Artisan Sourdoughs Over One Year of Daily Propagations Is Mainly Driven by Flour Microbiota and Nutrients
Source: Front Microbiol. 2018 Aug 27;9:1984. doi: 10.3389/fmicb.2018.01984 (PMC6119722; doi:10.3389/fmicb.2018.01984)
Supplement: Supplementary file 13 [file Presentation_2.PPTX]

## Slide 1
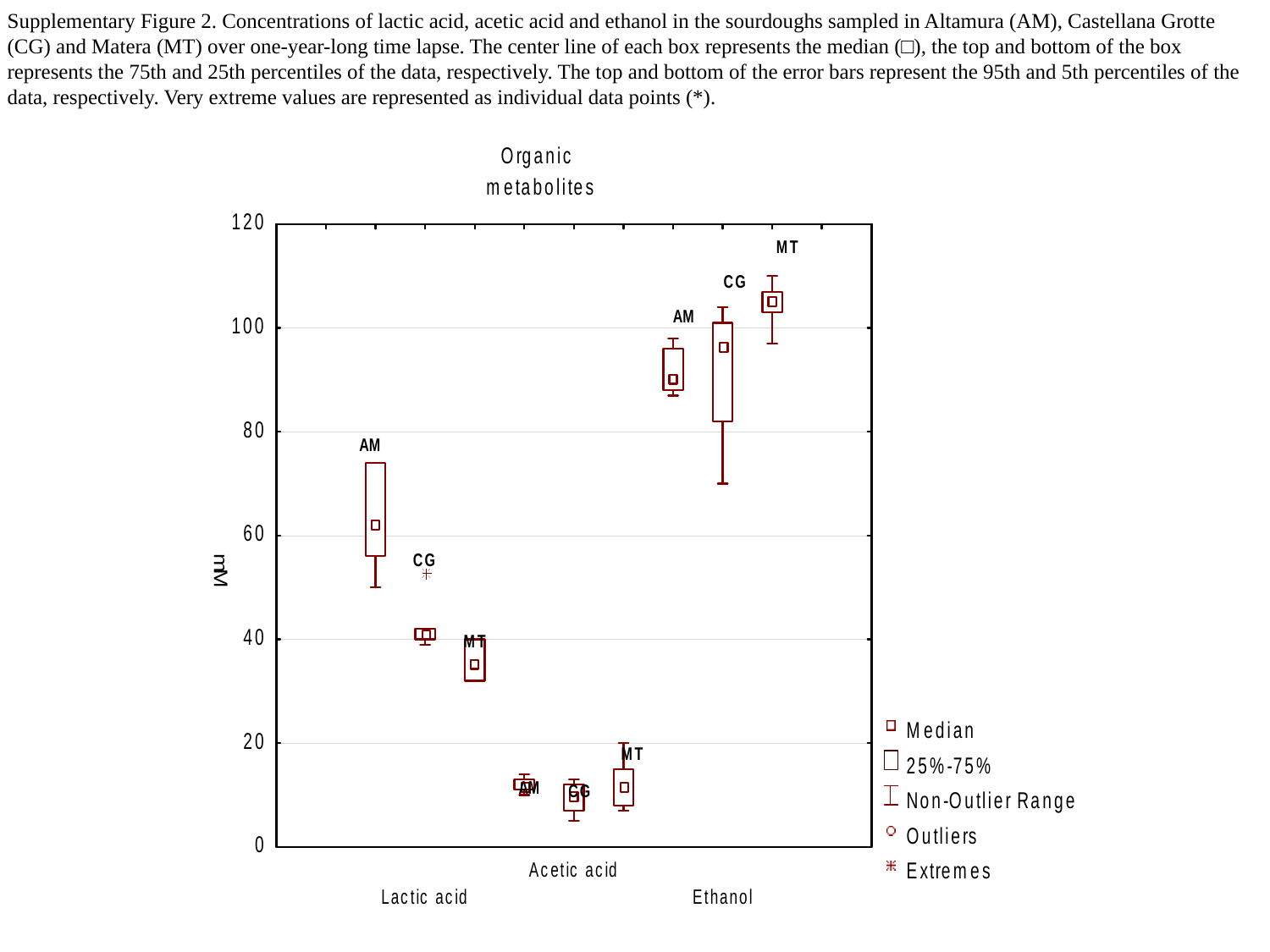

Supplementary Figure 2. Concentrations of lactic acid, acetic acid and ethanol in the sourdoughs sampled in Altamura (AM), Castellana Grotte (CG) and Matera (MT) over one-year-long time lapse. The center line of each box represents the median (□), the top and bottom of the box represents the 75th and 25th percentiles of the data, respectively. The top and bottom of the error bars represent the 95th and 5th percentiles of the data, respectively. Very extreme values are represented as individual data points (*).
